# Supplementary material for: Membrane-Proximal Epitope Facilitates Efficient T Cell Synapse Formation by Anti-FcRH5/CD3 and Is a Requirement for Myeloma Cell Killing
Source: Cancer Cell. 2017 Mar 13;31(3):383–95. doi: 10.1016/j.ccell.2017.02.001 (PMC5357723; doi:10.1016/j.ccell.2017.02.001)
Supplement: Document S1. Supplemental Experimental Procedures and Figures S1–S6 [file mmc1.pdf]

Cancer Cell, Volume *31*

Supplemental Information

Membrane-Proximal Epitope Facilitates Efficient T Cell Synapse Formation by Anti-FcRH5/CD3 and Is a Requirement for Myeloma Cell Killing

Ji Li, Nicola J. Stagg, Jennifer Johnston, Michael J. Harris, Sam A. Menzies, Danielle DiCara, Vanessa Clark, Maria Hristopoulos, Ryan Cook, Dionysos Slaga, Rin Nakamura, Luke McCarty, Siddharth Sukumaran, Elizabeth Luis, Zhengmao Ye, Thomas D. Wu, Teiko Sumiyoshi, Dimitry Danilenko, Genee Y. Lee, Klara Totpal, Diego Ellerman, Isidro Hötzel, John R. James, and Teemu T. Junttila

Supplemental Data

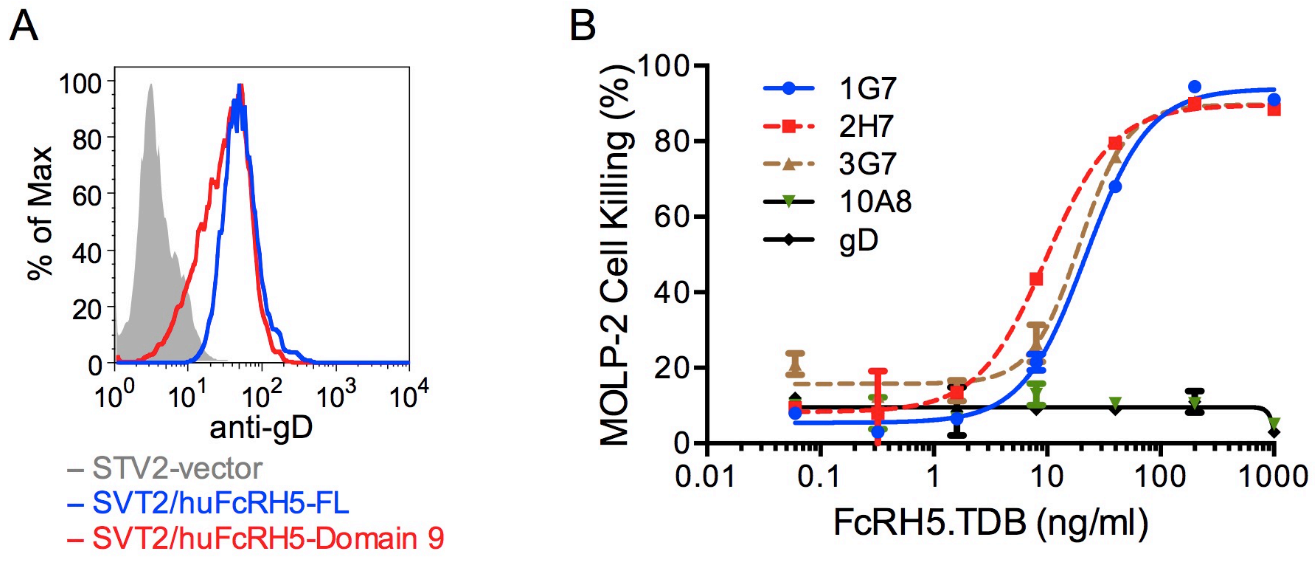

Figure S1. Expression level of full length FcRH5 or truncated FcRH5 in SVT2 cell lines and in vitro activity of multiple TDBs targeting membrane proximal region of the FcRH5. Related to Figure 2. (A) Histogram overlay of flow cytometry of SVT2-parental cells (grey), gD-FcRH5 full length (blue) and gD-FcRH5-domain 9 (red). (**B)** Activity of multiple TDBs targeting FcRH5 on MOLP2 cells. 10A8-TDB binds to distal regions of the endogenously expressed FcRH5. 1G7, 2H7 and 3G7-TDBs all bind to the membrane proximal region of the FcRH5. Data are represented as the mean ± SD.

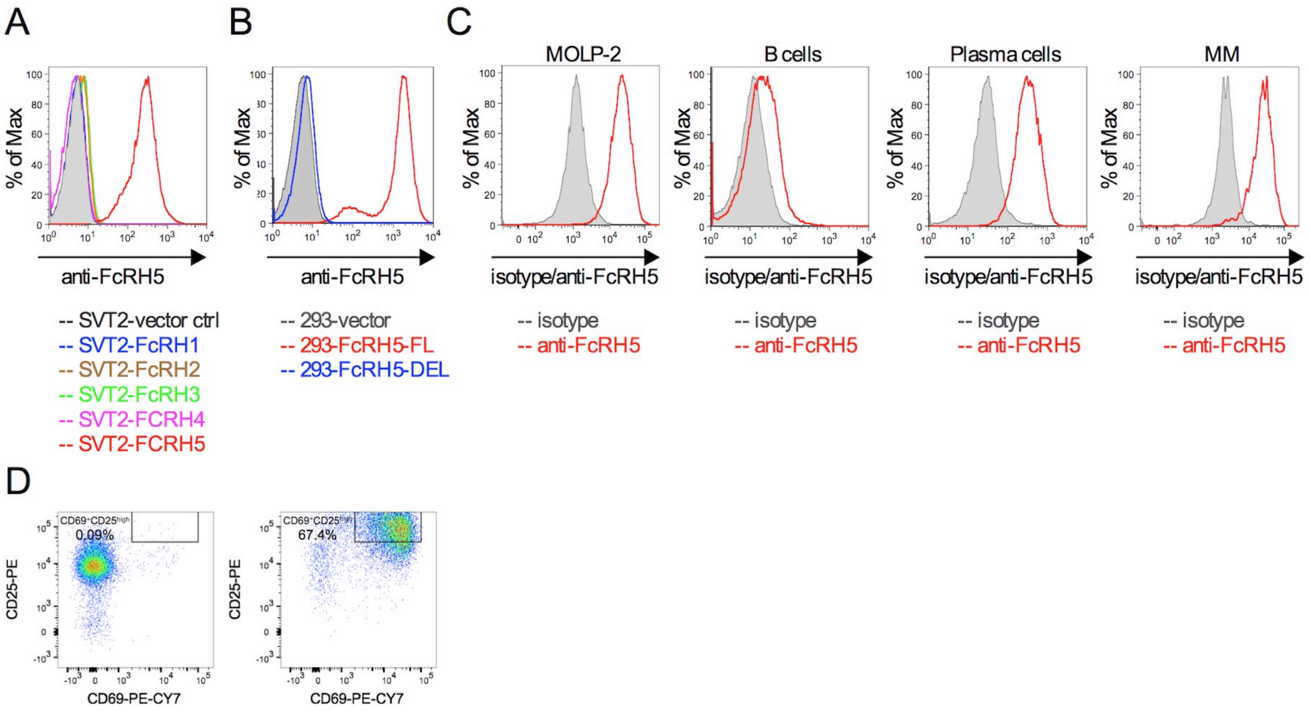

Figure S2. Binding specificity of anti-FcRH5 antibody clone 1G7 and activation of T reg cells by anti-FcRH5/CD3 TDB. Related to Figure 3. (A) Binding of clone 1G7 to FcRH family members. Histogram overlay of 6 cell lines, SVT2-vector (grey), SVT2-FcRH1 (blue), SVT2-FCRH2 (brown), SVT2-FcRH3 (green), SVT2-FcRH4 (purple), and SVT2-FcRH5 (red). **(B)** Histogram overlay of 1G7 binding to 3 cell lines, 293 parental (grey), 293-FcRH5 FL (full length; red) and 293-FcRH5-DEL (Deletion of AA464-850; blue). **(C)** Binding of clone 1G7 to MM cell line and primary cells. Overlay histogram of isotype-PE (grey) and anti-FcRH5 clone 1G7-PE (red) on MOLP-2 cell line, human $CD20^+$ B cells, human $CD38^+CD138^+$ plasma cells and $CD38^+CD138^+$ MM tumor cells from MM bone marrow aspirate. **(D)** Flow cytometry dot plots of T reg activation after 48 hr co-culture of sorted T regs and MOLP-2 in the absence of FcRH5/CD3 TDB (left) or in the presence of 1000 ng/ml of FcRH5/CD3 TDB (right). Activated T regs were classified as $CD69^+CD25^{high}$.

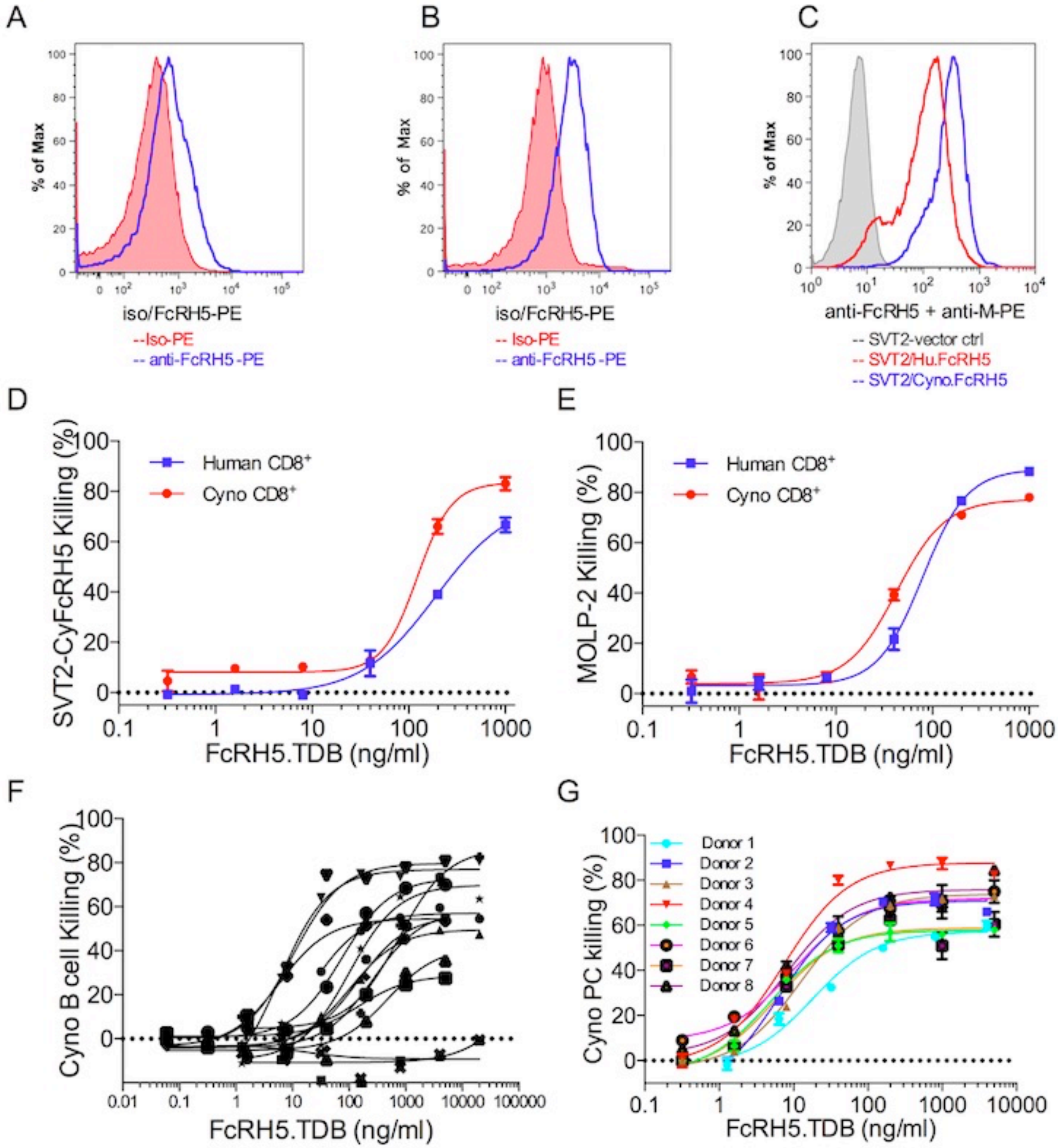

Figure S3. Cynomolgus monkey is an appropriate safety and efficacy model for anti-FcRH5/CD3. Related to Figure 7. TDB. Expression of FcRH5 on cyno CD20$^+$ B cells **(A)** and on CD45$^-$CD20$^-$CD38$^+$PC$^+$ cyno plasma cells **(B)**. Histogram overlays of isotype-PE (red) and anti-FcRH5 clone 1G7-PE (blue). **(C)** Binding of clone 1G7 to cyno FcRH5. Overlay histogram of 3 cell lines, SVT2-vector (grey), SVT2-hu.FcRH5 (red) and SVT2-cyno.FcRH5 (blue). **(D, E)** Cytotoxic activity of cyno CD8$^+$ T cells compared to human CD8$^+$ T cells. In vitro killing dose response on SVT2-cyno-FcRH5 **(D)** and MOLP-2 **(E)** with human CD8$^+$ T cells (blue) or cyno CD8$^+$ T cells (red). **(F)** In vitro killing activity of Anti-FcRH5/CD3 on cyno CD20$^+$ B cells (n=14). **(G)** In vitro killing activity of anti-FcRH5/CD3 on CD45$^-$CD20$^-$CD38$^+$PC$^+$ plasma cells from cyno bone marrow (n=8). Data are represented as the mean ± SD.

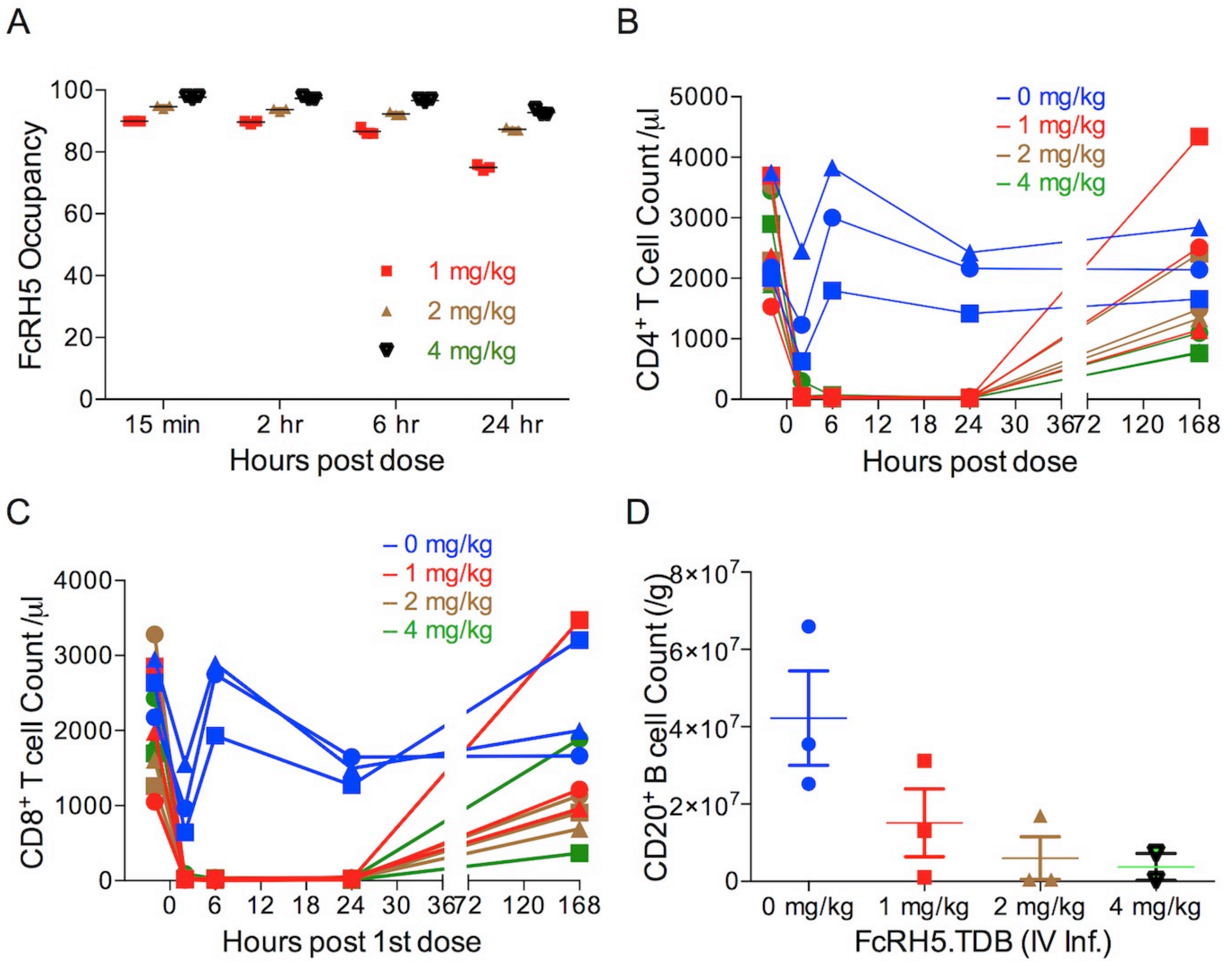

Figure S4. FcRH5 target occupancy, T cell dynamics ad depletion of B cells in mesenteric lymph nodes in cynomolgus monkey. Related to Figure 7. Four groups of animals after single dose intravenous administration of vehicle (blue), anti-FcRH5/CD3 at 1 mg/kg (red), 2 mg/kg (brown) or 4 mg/kg (green) were included in the graphs. **(A)** FcRH5 occupancy (RO) for anti-FcRH5/CD3 on circulating cyno B cells was calculated using a formula: % RO = C /(K_d + C) x 100. **(B)** The absolute count of $CD4^+$ T cells in peripheral blood. **(C)** The absolute count of $CD8^+$ T cells in peripheral blood. **(D)** Absolute counts of $CD20^+$ B cells in mesenteric lymph nodes after treatment. The plot is graphed as individual animals and the mean ± SEM.

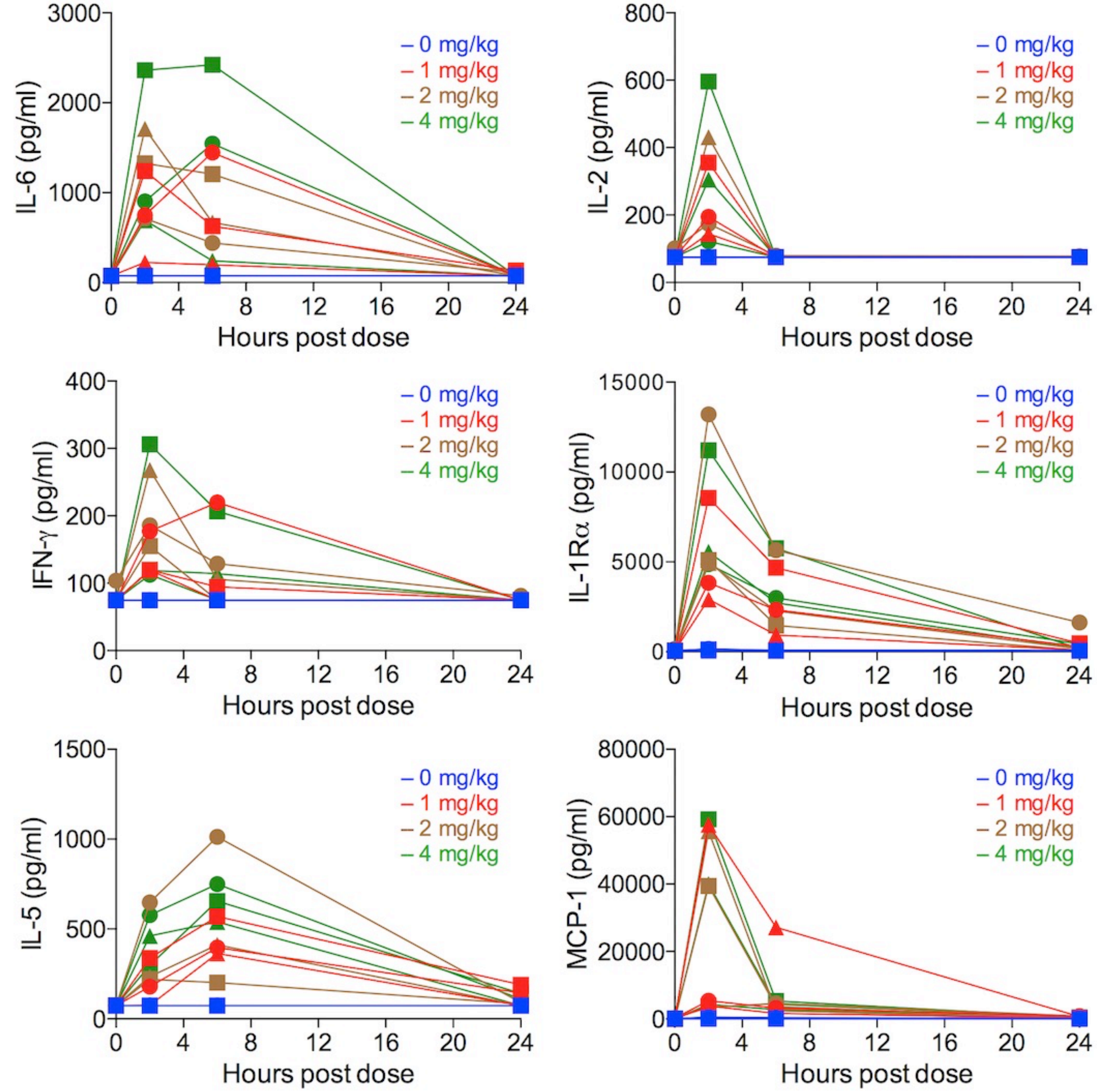

Figure S5. Anti-FcRH5/CD3 TDB induced transient cytokine release in cynomolgus monkey. **Related to Figure 7**. Four groups of animals after single dose intravenous administration of vehicle (blue), Anti-FcRH5/CD3 at 1 mg/kg (red), Anti-FcRH5/CD3 at 2 mg/kg (brown) and Anti-FcRH5/CD3 at 4 mg/kg (green) were included in the graphs.

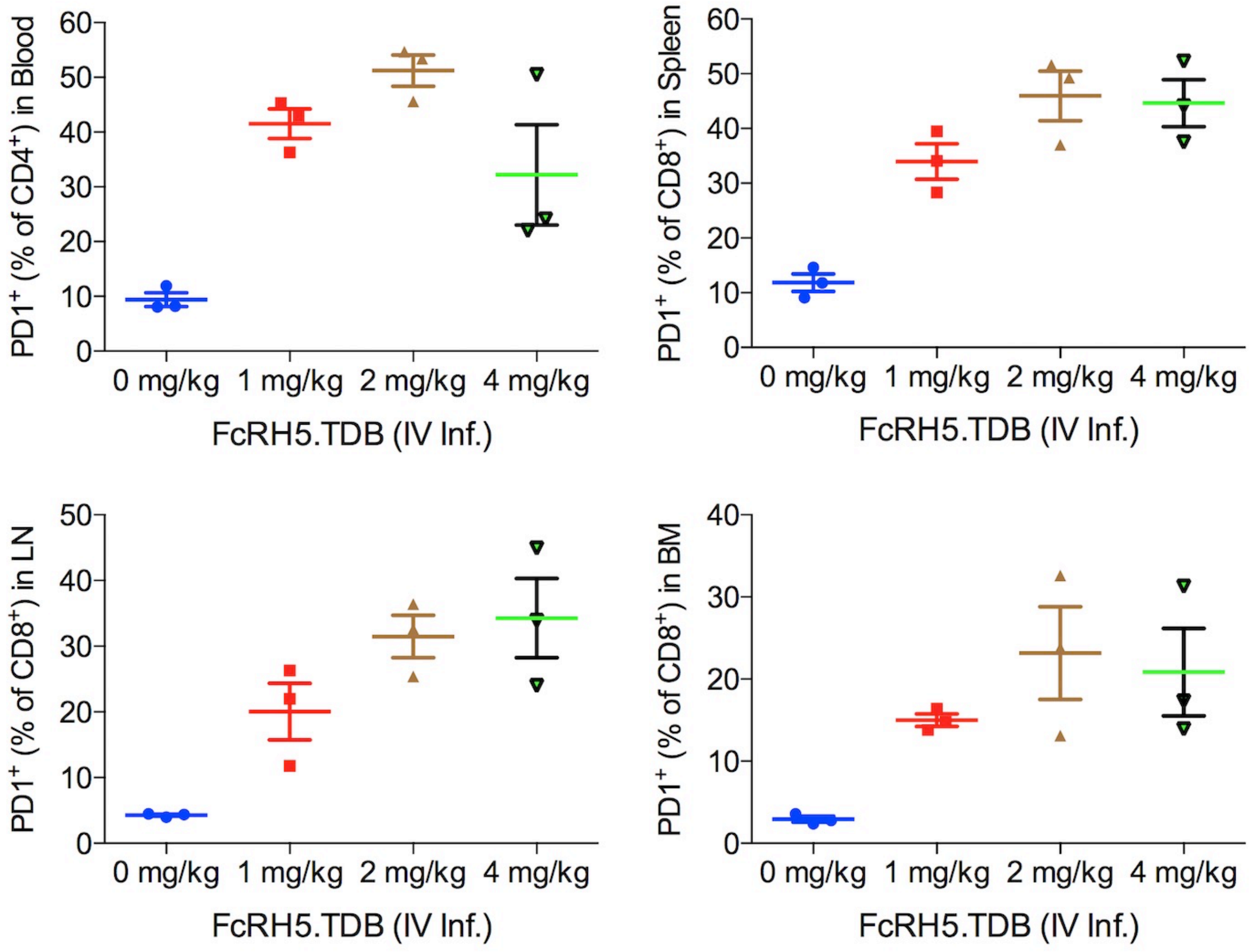

Figure S6. Anti-FcRH5/CD3 TDB induces PD-1 expression in cyno T cells in blood, spleen, lymph node and bone marrow. Related to Figure 8. PD-1 expression in $CD4^+$ and $CD8^+$ T cells was analyzed by flow cytometry 7 days after dosing anti-FcRH5/CD3 TDB or vehicle. Data are represented as the mean ± SEM.

Supplemental Experimental Procedures

RNA isolation, cDNA synthesis, and gene expression analysis

Total RNA was extracted from decalcified Formalin-fixed, paraffin-embedded (FFPE) bone marrow biopsy tissues collected from Multiple Myeloma patients. FFPE sections were deparaffinized, then lysate and RNA were prepared using the High Pure FFPET RNA Isolation Kit (Roche Applied Sciences, Indianapolis, IN) according to the manufacturer's protocol. Reverse transcription of complementary DNA (cDNA) and pre-amplification was generated in a single reaction using SuperScript® III Platinum® One-Step qRT-PCR Kit (ThermoFisher Scientific). Gene expression analysis was performed on the Fluidigm Biomark HD with custom Taqman assays (Applied Biosystems). The thermal cycling conditions are as follows: 1 cycle at 50˚C for 15 min, 1 cycle at 70˚C for 2 min, then 14 cycles at 95˚C for 15 sec and 60˚C for 4 min. Two reference genes, SDHA and VPS33B, were evaluated for each sample and used to calculate expression of FcRH5. Gene expression of FcRH5 was determined by using the delta Ct (dCt) method [$Ct_{\text{gene of interest}} - Ct_{\text{geometric mean of reference genes}}$].

Cytogenetic fluorescent in situ hybridization (FISH)

1q21 + copy control 1 FISH probe (BioCare Medical; previously CymogenDx, Concord, CA) was used to analyze 1q21 region. The 1q21 probe covers the chromosomal band 1q21.3 while the control probe is located in the peri-centromeric 1p12 region of chromosome 1. FISH analysis on formalin-fixed paraffin-embedded (FFPE) tissue was performed as described previously (Koeppen et al., 2014; O'Brien et al., 2008) with some modifications. After an overnight incubation at 56°C, the FFPE slides (5μ thick) were de-paraffinized in 3 washes of CitroSolv for 5 min each, dehydrated by two washes in alcohol and air-dried. Subsequently, the FFPE sections were incubated in a Pretreatment solution (Abbott Molecular) for 30 min at 80ºC and followed with a Protease II (Abbott Molecular) digestion prior to additional washes in water and a series of ethanol. Dried slides were then co-denatured (76°C for 6 min) with the probe set and were hybridized overnight at 37°C (ThermoBrite; Vysis, Downers Grove, IL). Post-hybridization washes and counter- staining were done in a manner similar to those previously described (Koeppen et al., 2014). The sections were viewed using fluorescence microscopes equipped with computer-driven imaging systems (BioView Duet, BioView Ltd., Israel). In the FFPE tissue samples, a minimum of 100 non-overlapping tumor cells from each sample was enumerated for the 1q21.3 (target) and 1p12 (control) locus. A tumor sample was identified as FISH positive when >20% of the tumor cells scored had 3 or more copies of the 1q21.3 locus.

huNSG/MOLP-2 mouse xenograft model

Since anti-CD3 antibodies do not cross-react with mouse CD3 we used a mouse model with reconstituted human immune systems ("humanized mice") to demonstrate in vivo anti myeloma activity of anti-FcRH5/CD3 TDB. Irradiated female NOD.Cg Prkdcscid Il2rgtm1Wjl/SzJ (common name NOD/scid gamma; NSG) mice were transplanted with CD34+ selected human hematopoietic stem cells to generate the humanized huNSG mice. huNSG mice were obtained from The Jackson Laboratory. 20 weeks post CD34+ cell transplantation huNSG mice were inoculated with 20 million MOLP-2 cells subcutaneously. MOLP-2 (American Type Culture Collection, Manassas, VA) is a human multiple myeloma cell line that express human FcRH5 endogenously and can be used as a xenograft tumor model. On the day of the cell inoculation, 5 animals were inoculated with 0.2 ml of MOLP2 tumor cells at a concentration of 100 million cells/ml, in HBSS/matrigel (1:1 ratio), subcutaneously in the right flank. As soon as the tumor volumes reached a volume range of 100-250 mm^3, animals were randomized into 2 groups and the first treatments were administered at that time (Day 0). All treatments were administered once a week for by intravenous (IV) tail vein injection. Tumors were measured 1-2 times per week with calipers for the duration of the study and animal body weights were recorded at least once a week. Clinical observations were performed twice per week to monitor the health of the animals. All in vivo experimental procedures conformed to the guiding principles of the American Physiology Society and were approved by Genentech's Institutional Animal Care and Use Committee. Only animals that appeared to be healthy and that were free of obvious abnormalities were used for the study.
