# Supplementary material for: Membrane-Proximal Epitope Facilitates Efficient T Cell Synapse Formation by Anti-FcRH5/CD3 and Is a Requirement for Myeloma Cell Killing
Source: Cancer Cell. 2017 Mar 13;31(3):383–95. doi: 10.1016/j.ccell.2017.02.001 (PMC5357723; doi:10.1016/j.ccell.2017.02.001)
Supplement: Document S2. Article plus Supplemental Information [file mmc2.pdf]

Article

Cancer Cell

Membrane-Proximal Epitope Facilitates Efficient T Cell Synapse Formation by Anti-FcRH5/CD3 and Is a Requirement for Myeloma Cell Killing

Highlights

- Prevalence of FcRH5 expression in multiple myeloma is 100%
- Anti-FcRH5/CD3 TDB redirects T cells to kill myeloma cells
- Target clustering and CD45 exclusion activate T cells
- Anti-FcRH5/CD3 TDB is a highly efficacious immunotherapy for myeloma

Authors

Ji Li, Nicola J. Stagg,
Jennifer Johnston, ..., Isidro Hötzel,
John R. James, Teemu T. Junttila

Correspondence

junttila.teemu@gene.com

In Brief

Li et al. report that the size and epitope location of the target play a key role in the efficiency of T cell activation induced by T cell-dependent bispecific antibodies (TDBs). They develop a TDB targeting FcRH5 expressed in all multiple myeloma tumor cells and show its potential in treating this disease.

Li et al., 2017, Cancer Cell *31*, 383–395
March 13, 2017 © 2017 Genentech. Published by Elsevier Inc.
http://dx.doi.org/10.1016/j.ccell.2017.02.001

Cancer Cell
Article

CellPress

Membrane-Proximal Epitope Facilitates Efficient T Cell Synapse Formation by Anti-FcRH5/CD3 and Is a Requirement for Myeloma Cell Killing

Ji Li,[1] Nicola J. Stagg,[1] Jennifer Johnston,[1] Michael J. Harris,[2] Sam A. Menzies,[2] Danielle DiCara,[1] Vanessa Clark,[1] Maria Hristopoulos,[1] Ryan Cook,[1] Dionysos Slaga,[1] Rin Nakamura,[1] Luke McCarty,[1] Siddharth Sukumaran,[1] Elizabeth Luis,[1] Zhengmao Ye,[1] Thomas D. Wu,[1] Teiko Sumiyoshi,[1] Dimitry Danilenko,[1] Genee Y. Lee,[1] Klara Totpal,[1] Diego Ellerman,[1] Isidro Hötzel,[1] John R. James,[2] and Teemu T. Junttila[1,3,*]

[1]Genentech, Inc., 1 DNA Way, South San Francisco, San Francisco, CA 94080, USA
[2]Molecular Immunity Unit, Department of Medicine, University of Cambridge, MRC-LMB, Cambridge, CB2 0QH, UK
[3]Lead Contact
*Correspondence: junttila.teemu@gene.com
http://dx.doi.org/10.1016/j.ccell.2017.02.001

SUMMARY

The anti-FcRH5/CD3 T cell-dependent bispecific antibody (TDB) targets the B cell lineage marker FcRH5 expressed in multiple myeloma (MM) tumor cells. We demonstrate that TDBs trigger T cell receptor activation by inducing target clustering and exclusion of CD45 phosphatase from the synapse. The dimensions of the target molecule play a key role in the efficiency of the synapse formation. The anti-FcRH5/CD3 TDB kills human plasma cells and patient-derived myeloma cells at picomolar concentrations and results in complete depletion of B cells and bone marrow plasma cells in cynomolgus monkeys. These data demonstrate the potential for the anti-FcRH5/CD3 TDB, alone or in combination with inhibition of PD-1/PD-L1 signaling, in the treatment of MM and other B cell malignancies.

INTRODUCTION

Multiple myeloma (MM) is an incurable malignancy of plasma cells characterized by dysregulated growth of abnormal plasma cells in the bone marrow and overproduction of intact monoclonal immunoglobulins that ultimately lead to clinical manifestations including skeletal lesions, renal failure, anemia, and hypercalcemia. Currently the backbone of MM treatment involves combinations of proteasome inhibitors (PIs), immunomodulators, and corticosteroids, with bone marrow transplantation as an additional option for eligible patients. Newer agents are being developed for the treatment of MM, including the monoclonal antibodies targeting CD38 (daratumomab) and SLAMF7 (elotuzumab). Nevertheless, despite progressive improvements in myeloma treatment, the mortality rate remains high and median survival remains less than 5 years (http://seer.cancer.gov/).

Heterogeneous disease biology and genetics, limited availability of predictive preclinical models, and a paucity of known myeloma-specific surface targets remain key challenges in myeloma drug development. FcRH5 (also known as FcRL5, IRTA2, or CD307) has been identified as an attractive B cell lineage-specific surface marker in myeloma (Elkins et al., 2012; Hatzivassiliou et al., 2001; Polson et al., 2006). High-affinity ligands and biological significance of FcRH5 are largely unknown. FcRH5 is expressed exclusively in the B cell lineage. Expression is detected as early as pre-B cells (Polson et al., 2006); however, unlike other B cell-specific surface proteins (e.g., CD20, CD19, and CD22), FcRH5 expression is retained in plasma cells. Analogous to its expression in normal plasma cells, FcRH5 is expressed by myeloma tumor cells. Finally, FcRH5 expression has been reported in several B cell malignancies (Ise et al., 2007; Li et al., 2008; Polson et al., 2006), suggesting broader applicability of this target in hematological malignancies.

Therapies that direct T cells to tumors, including adoptive transfer of genetically engineered T cells and T cell-dependent bispecific antibodies (TDBs) that selectively recruit T cells to tumor cells have been clinically validated in the treatment of B cell leukemias and lymphomas (Bargou et al., 2008; Sadelain, 2015)

Significance

Our study describes how CD3-bispecific antibody “triggers” intracellular T cell signaling and shows that the dimensions of the target molecule and epitope location play a key role in the efficiency of the synapse formation and subsequent T cell activation. These findings are important for future design of T cell-recruiting therapies. Using this information we developed and preclinically validated an anti-FcRH5/CD3 TDB as an immunotherapy for multiple myeloma. The anti-FcRH5/CD3 TDB is highly efficacious in the killing of myeloma cells and depletes bone marrow plasma cells in primates.

This is an open access article under the CC BY license (http://creativecommons.org/licenses/by/4.0/).

and have demonstrated promising activity in myeloma (Garfall et al., 2015; Rapoport et al., 2015). Our previous preclinical studies have validated full-length bispecific antibodies as an optimal TDB format with favorable drug-like properties including long serum half-life and low risk for anti-drug antibodies (Junttila et al., 2014; Sun et al., 2015).

TDBs activate T cells upon ligation with target-expressing cells resulting in potent target cell killing. However, the molecular mechanism that induces T cell activation has not been described in detail. The close proximity of cell membranes forms the basis of the kinetic segregation model for T cell receptor (TCR) triggering (Davis and van der Merwe, 2006). The model proposes that the exclusion of inhibitory molecules, such as CD45 phosphatase, from regions of close cell-cell apposition causes increased kinase activity and leads to phosphorylation of peptide-major histocompatibility complex (pMHC)-bound TCRs within this region. This then initiates receptor triggering and subsequent downstream T cell activation. Exclusion of CD45 from the synapse has been shown to be a passive process driven by the large size of the extracellular domain (James and Vale, 2012). If correct, the model predicts that a tumor target with a large extracellular domain may be suboptimal for synapse formation by CD3-bispecific antibodies. The size of the target protein has previously been linked to the killing activity of bispecific T cell engagers (BiTE) (Bluemel et al., 2010).

Given the potential of FcRH5 as a target for antibody-based therapeutics, the goal of the current study was to develop a TDB targeting FcRH5 (anti-FcRH5/CD3 TDB) for the treatment of MM. Moreover, we characterized the molecular events in the immunological synapse that lead to triggering of the TCR upon stimulation by CD3-bispecific antibodies.

RESULTS

Anti-FcRH5/CD3 TDB Induces Target Clustering and Exclusion of CD45 from the Synapse Resulting in TCR Triggering

We characterized the molecular events in the immunological synapse that lead to triggering of the TCR upon stimulation by CD3-bispecific antibodies. To do this, we utilized a recently described reconstituted system that allows investigation of the initial events that lead to receptor activation in a controlled manner. HEK-T cells are non-immune cells that express the minimal components of the TCR signaling network required to drive receptor activation (James and Vale, 2012). Previous studies using this model have demonstrated that exclusion of CD45 phosphatase from the cell-cell interphase is both necessary and sufficient for TCR-pMHC-driven TCR triggering, supporting the kinetic segregation model (James and Vale, 2012). The HEK-T cells were conjugated with FcRH5-expressing cells in the presence of the CD3 bispecific antibody, and the relative intensities of CD45, FcRH5, and the fluorescently labeled TDB at the conjugate interface were imaged by confocal microscopy. ZAP70 is normally cytosolic but binds to phosphorylated ITAMs on the TCR when the receptor is bound to pMHC. The translocation of ZAP70 provides a convenient microscopy-based assay to evaluate TCR triggering (James and Vale, 2012). TDB binding to the membrane-proximal immunoglobulin (Ig) domain of FcRH5 (1G7; Figure 1A) led to efficient synapse formation, a robust clustering of FcRH5 in the cell-cell interaction site, and exclusion of the CD45 from the synapse (Figure 1B). The combined result of FcRH5 clustering and exclusion of inhibitory molecules was TCR triggering illustrated by ZAP70 translocation to the cell interface (Figure 1B).

We then compared the sequence of events in synapse formation at the cell-cell interface when driven by CD3-bispecific antibody binding (Figures 1A and 1B) with those at the interface caused by the pMHC-TCR interaction itself (Figures 1C and 1D). Overall, the mechanisms leading to TCR triggering, including target clustering, CD45 exclusion, and ZAP70-translocation, showed remarkable similarity between the CD3-bispecific antibody and pMHC-driven ligation (Figures 1B and 1D).

The interface between the two cells can be visualized by taking a three-dimensional volume of the cell-cell conjugate formed by the TDB (Figure 1E). The analysis demonstrated that the segregation of CD45 and the concomitant clustering of FcRH5 caused by TDB binding extended across the entire interface. The analysis also demonstrated a spatial mutual exclusion of the FcRH5 and the CD45 phosphatase. To confirm that this was the case, we analyzed a line profile across an equivalent interface and quantified the relative intensities of CD45, FcRH5, and the TDB (Figure 1F). The quantitation of the fluorescent signal confirmed the strong co-localization between FcRH5 and the TDB, and the complete inverse correlation with CD45 fluorescence intensity. In summary, our results demonstrate that the CD3-bispecific antibody replicates the mechanism of the TCR/pMHC interaction-mediated TCR triggering inducing clustering of the target molecule and exclusion of CD45 from the T cell synapse resulting in activation of TCR signaling. CD45 exclusion has been described in the synapse induced by an Ep-CAM/CD3 BiTE (Offner et al., 2006), demonstrating that the conjugate interface and the molecular mechanism leading to T cell triggering share common features despite distinct structures of these molecules.

A Membrane-Proximal Epitope Is Required for Efficient Synapse Formation and Killing Activity of the Anti-FcRH5/CD3 TDB

Similar to CD45, the extracellular domain of FcRH5 is large (550 and 835 amino acids, respectively) allowing us to test the effect of structural parameters of the tumor target on synapse formation and killing activity. We generated three proof-of-concept TDBs that bind to different regions of FcRH5 (Figure 2A). TDB binding to the membrane-proximal Ig domain of FcRH5 (1G7) led to synapse formation significantly more efficiently compared with TDBs targeting central (10A8) or distal domains (gD) of the target (Figure 2B), driving efficient CD45 exclusion (Figures 2B and 2C) and target clustering (Figures 2B and 2D) at the cell-cell interface. The efficiency of forming a synapse was reflected in the ability of the TDB to induce TCR signaling and mediate killing by human T cells. Using healthy donor $CD8^+$ T cells, 1G7-TDB resulted in very robust SLP76 phosphorylation (Figure 2E), which is indicative of potent TCR signaling, and mediated efficient killing of target cells (Figure 2F; median effective concentration (EC_{50} = 0.5 nM). In contrast, gD-TDB targeting of a membrane-distal epitope resulted in undetectable TCR signaling and was unable to mediate T cell killing. Monovalent binding affinities of antibodies used (K_D = 12 versus 3 nM by

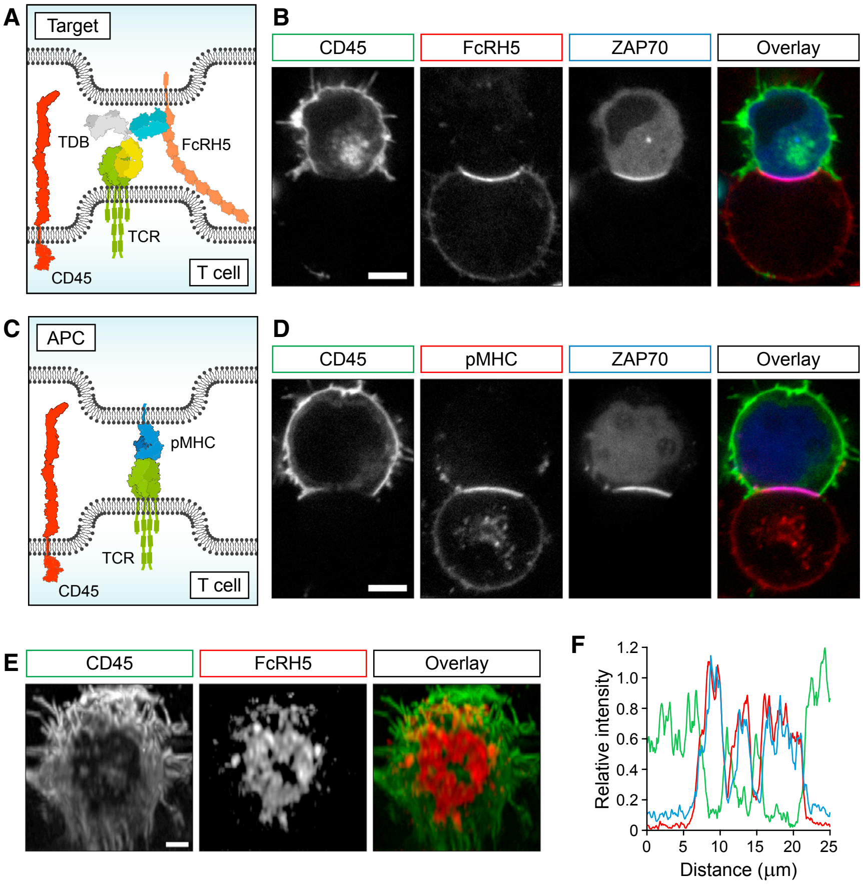

Figure 1. Anti-FcRH5/CD3 TDB Induces Target Clustering and Exclusion of CD45 from the Synapse Resulting in TCR Triggering

(A) Schematic of the interface between the FcRH5-expressing target cell and the reconstituted HEK-T cell, with protein dimensions shown approximately to scale. The remaining components of the HEK-T cells, including the normally cytoplasmic kinase, ZAP70, have been omitted for clarity.

(B) Representative images of TDB-mediated conjugates (1G7-TDB) between an FcRH5-expressing HEK cell and a reconstituted HEK-T cell, showing CD45 segregation, FcRH5 clustering, and ZAP70 recruitment at the cell interface.

(C) Schematic of the interface between the pMHC-expressing target cell and the reconstituted HEK-T cell, with protein dimensions shown approximately to scale. The remaining components of the cells, including the normally cytoplasmic kinase, ZAP70, have been omitted for clarity.

(D) Representative images of conjugates between a pMHC-expressing Raji B cell and a reconstituted HEK-T cell, showing CD45 segregation, pMHC clustering, and ZAP70 recruitment at the cell interface.

(E) A reconstructed en face view of a conjugate interface equivalent to that shown in (B).

(F) Line profiles of CD45 (green), FcRH5 (blue), and TDB (red) intensities across a conjugate interface equivalent to that shown in (B). In all images, box color denotes that used in the overlay image.

Scale bars, 5 μm (B) and (D) or 2 μm (E) in length.

Scatchard analysis for 1G7 and 10A8, respectively) do not explain the differences in the synapse formation or signaling.

We next confirmed that the TDB activity is driven primarily by the location of the epitope and the size of the extracellular domain (ECD) by targeting cells that express a heavily truncated FcRH5 that retains the 1G7 and gD epitopes (Figure 2G). The activity of the proximal 1G7-TDB increased by 25-fold (Figure 2H; EC_{50} = 20 pM) and the gD-TDB was able to effectively mediate killing of cells (EC_{50} = 0.19 nM) when the obstruction caused by the ECD was removed. The possibility of differential target expression level being the cause for the activity difference between cell lines was ruled out by flow cytometry (Figure S1A). To confirm that the differences in the killing activity are related to the epitope rather than being properties of the specific TDB clones, we tested a total of five unique antibody clones targeting the membrane-proximal domain for the FcRH5 in bispecific format and could demonstrate that the activity of each clone was ~20-fold higher compared with 10A8 (Figure 2I). These findings were confirmed using the MOLP-2 myeloma cell line, which endogenously expresses FcRH5 at a low level comparable with that of MM patients. When T cells were retargeted to kill MOLP-2 cells, only membrane-proximal TDBs induced killing of the MOLP-2 cells. Targeting the mid region of FcRH5 would not lead to sufficiently potent TCR triggering to kill MOLP-2 cells (Figure S1B). Together these data demonstrate that formation of the immunologic synapse by a CD3-bispecific antibody is dependent on the dimensions of the target molecule, and that rational epitope selection based on membrane proximity can overcome the hindrance caused by a large target protein that would otherwise lead to suboptimal synapse formation.

The Anti-FcRH5/CD3 TDB Induces Target-Dependent Cell Killing and T Cell Proliferation

The anti-FcRH5 clone 1G7 was humanized and paired with a humanized anti-CD3 arm that is cross-reactive to the cynomolgus monkey to generate an anti-FcRH5/CD3 TDB. Anti-FcRH5/CD3 TDB is specific to FcRH5 and binds to the membrane-proximal domain of the target (Figures S2A–S2B). Anti-FcRH5/CD3 TDB binds to FcRH5-expressing myeloma cell lines (MOLP-2), healthy donor B cells, bone marrow plasma cells, and primary myeloma tumor cells (Figure S2C). Preclinical characterization of anti-FcRH5/CD3 TDB activity to confirm its mechanism of action was performed. Treatment of FcRH5-positive MOLP-2 cells with the anti-FcRH5/CD3 TDB and $CD8^+$ or $CD4^+$ T cells from healthy donors resulted in dose-dependent T cell activation and killing of MOLP-2 cells (Figures 3A and 3B). Anti-FcRH5/CD3 TDB also had an effect on T_{reg} cell activation (Figure S2D). The cytotoxic activity of TDB required FcRH5 expression on target cells and the potency correlated with FcRH5 expression level (Figure 3C). Stimulation of effector T cells with the anti-FcRH5/CD3 TDB in the presence of target cells led to a robust proliferation of T cells, with 95% of the $CD8^+$ cells undergoing as many as six cell divisions in 5 days (Figure 3D).

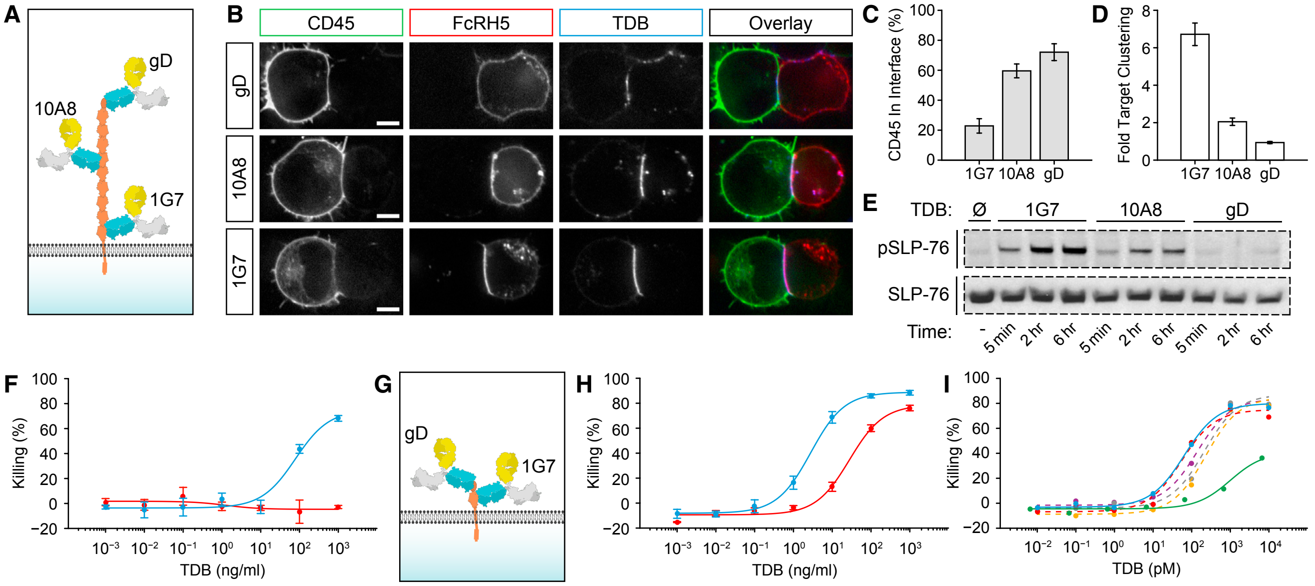

Figure 2. Membrane-Proximal Epitope Is Required for Efficient Synapse Formation and Killing Activity of Anti-FcRH5/CD3

(A) Schematic of the epitope locations of the three TDBs used in the figure, including the gD epitope at the N terminus.

(B) Representative confocal images of the interface between a HEK-T cell and an FcRH5-expressing HEK cell, where conjugate formation has been induced using the indicated fluorescently labeled TDB. The overlay image identifies the cell-cell interface over which the intensities of CD45, FcRH5, and TDB were measured, relative to the rest of the plasma membrane. Box color denotes that used in the overlay image. Scale bars, 5 μm.

(C) Quantification of CD45 intensity in the conjugate interface relative to CD45 intensity in the rest of the plasma membrane. Data shown are the mean from 12 to 17 conjugates for each TDB is shown ±SEM.

(D) Equivalent quantification of FcRH5 intensity in the same cells used in (C), presented as fold-increase of intensity compared with rest of target plasma membrane ± SEM.

(E) Phospho-SLP76 western blot of peripheral CD8+ T cells from a healthy donor stimulated with 1 μg/mL TDBs and cells expressing human FcRH5 with an N-terminal gD expression tag. Total SLP76 is shown to confirm equal sample loading.

(F) FcRH5 target cell killing using either 1G7 (blue) or gD (red) TDBs using CD8+ T cells.

(G) Schematic of the truncated FcRH5 construct with the gD tag now proximal to the membrane.

(H) Target cell killing using the truncated FcRH5 construct using 1G7 (blue) or gD (red) TDBs. The truncated construct was expressed in HEK293 cells.

(I) Target cell killing using 1G7 TDB (blue), alternate TDBs that recognize the membrane-proximal epitope (dashed lines), or 10A8 TDB (green). Data in (F), (H), and (I) are represented as the mean ± SD. See also Figure S1.

Expression of FcRH5 in Normal Tissues and MM

FcRH5 is expressed in the B cell lineage starting from pre-B cells, but unlike most B cell markers, its expression is retained in plasma cells (Polson et al., 2006). Analysis of FcRH5 RNA expression in the Genotype-Tissue Expression (GTEx) sample set (Ardlie et al., 2015), consisting of 8,555 samples from 544 donors over 53 tissues, demonstrated expression in Epstein-Barr virus- transformed lymphocytes, spleen, and the terminal ileum of the small intestine (Figure 4A). The RNA signal detected in spleen and intestine is likely derived from infiltrating B cells. Further analysis demonstrated a strong correlation with the expression profile of known B cell and plasma cell markers (CD19, CD20, and BCMA; Figure 4B). Overall the selective expression in B lineage cells and tissues predicts a favorable safety profile for this target.

Expression of FcRH5 in CD138^{+}CD38^{+} MM cells and normal bone marrow plasma cells was evaluated by flow cytometry using bivalent 1G7 antibody. In all samples tested, all patient-derived tumor cells, and all normal plasma cells expressed FcRH5, suggesting 100% prevalence in myeloma (Figure 4C). Considerable inter-patient variability in expression level was detected in MM samples. Generally, FcRH5 expression levels in tumor cells was not significantly elevated compared with normal plasma cells, suggesting that developing a tumor cell-selective, normal plasma cell-sparing anti-FcRH5/CD3 TDB may not be feasible. FcRH5 expression level in normal B cells was observed to be lower compared with normal plasma cells and MM tumor cells (Figures 4C and S2C), a similar finding as a previous report (Elkins et al., 2012). To understand the prevalence of expression in a broader patient population we performed a bioinformatic analysis of FcRH5 mRNA expression in CD138-purified plasma cells from 170 non-treated newly diagnosed MM patients and 6 healthy donors (microarray dataset GSE39754 from the NCBI Gene Expression Omnibus). All myeloma samples were positive for FcRH5 RNA (Figure 4D). At the mRNA level, FcRH5 expression was significantly higher in myeloma samples compared with healthy donor samples. Expression of FcRH5 was higher in 155 out of 170 (91%) malignant samples compared with the highest expression level detected in the normal samples. Only one clinical value of interest, response to the USP7 inhibitor P5091, is available for the samples, and there was no significant correlation between this treatment response and FcRH5 expression ($p = 0.62$; ANOVA). The FcRH5 gene is located in the chromosomal breakpoint in 1q21 (Hatzivassiliou et al., 2001).

CellPress

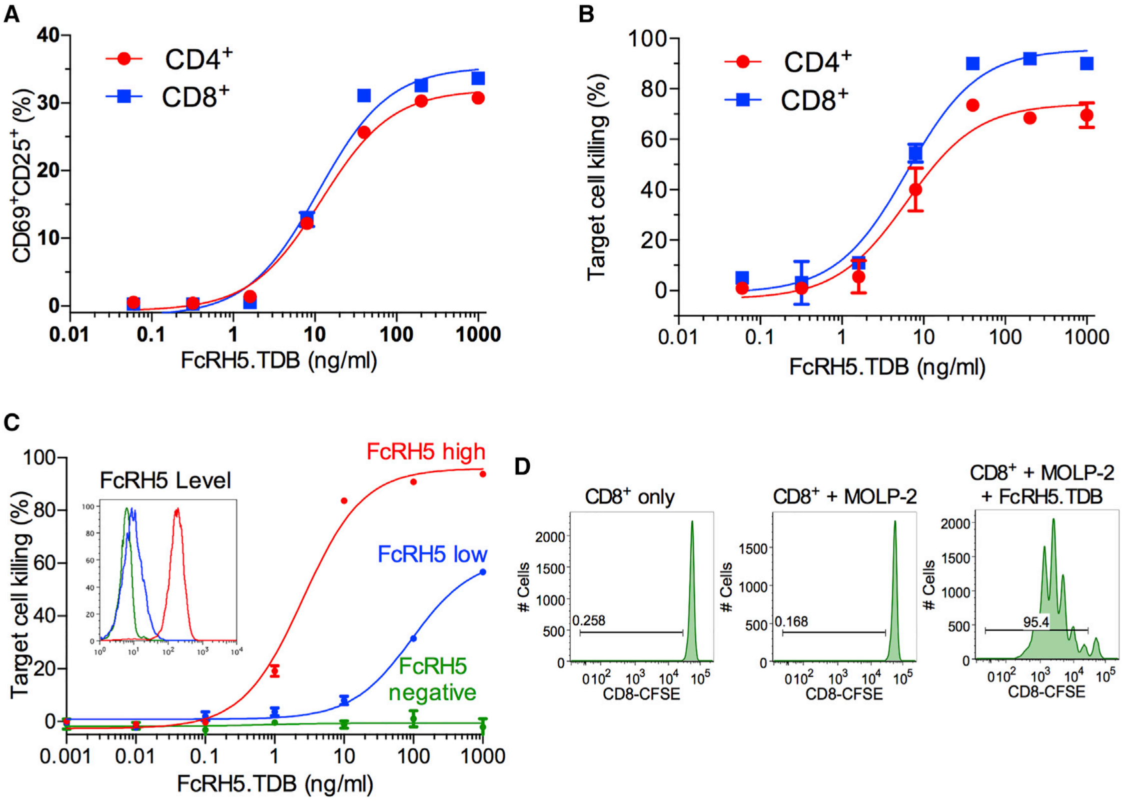

Figure 3. Anti-FcRH5/CD3 TDB Induces T Cell Activation, Target-Dependent Cell Killing, and T Cell Proliferation

(A) Dose-dependent activation of $CD4^+$ and $CD8^+$ T cells upon stimulation with target cells (MOLP-2) and anti-FcRH5/CD3 TDB detected by flow cytometry analysis.

(B) Target cell (MOLP-2) killing by anti-FcRH5/CD3 TDB and effector cells ($CD4^+$ or $CD8^+$).

(C) Target-dependent killing by anti-FcRH5/CD3 TDB and flow cytometry (insert) of a parental Fox-NY cell line (green) and clones transfected to express low (blue) or high level (red) of FcRH5. (A), (B), and (C) Data are represented as the mean ± SD.

(D) Anti-FcRH5/CD3 TDB-induced $CD8^+$ T cell proliferation response (5 days) was detected by measuring carboxyfluorescein succinimidyl ester (CFSE) fluorescence intensity dilution. Only CFSE-labeled human $CD8^+$ T cells and those co-cultured with MOLP-2 ± anti-FcRH5/CD3 TDB (1 μg/mL) are shown. See also Figure S2.

Analysis of ~20 primary MM biopsies demonstrated a significant association between FcRH5 RNA expression and 1q21 gain (Figures 4E–4F), demonstrating that the 1q21 gain can lead to FcRH5 overexpression in high-risk myeloma patients.

The selective expression for B lineage cells and tissues predicts a favorable safety profile for this target. Overall, 100% prevalence in myeloma, the predicted favorable safety profile and overexpression in high-risk patients indicate FcRH5 as a promising target for MM.

The Anti-FcRH5/CD3 TDB Mediates Potent Killing of Normal Plasma Cells and Patient-Derived Primary Myeloma Cells

The ability of the anti-FcRH5/CD3 TDB to kill plasma cells was analyzed by targeting bone marrow mononuclear cells (BMMCs) isolated from bone marrow aspirates of healthy donors (Figure 5A). Anti-FcRH5/CD3 TDB treatment induced potent dose-dependent killing of normal plasma cells (EC_{50} = 85–180 pM). Similarly robust cytotoxic activity was detected when BMMCs from MM patients were exposed to the anti-FcRH5/CD3 TDB (Figure 5B). The anti-FcRH5/CD3 TDB demonstrated near-complete and highly potent killing of primary myeloma tumor cells (EC_{50} = 60–1,200 pM).

As FcRH5 expression is variable in myeloma (Figures 4C–4D) and anti-FcRH5/CD3 activity correlated with expression level (Figure 3C), we investigated whether patients whose tumor cells expressed low levels of FcRH5 would be predicted to respond to the anti-FcRH5/CD3 TDB. The MOLP-2 myeloma cell line was identified as a benchmark cell line that has similar expression levels of FcRH5 as plasma cells and primary MM cells (Figure 4C). We also identified additional cell lines that express extremely low levels of FcRH5 and determined the number of FcRH5 molecules per cell using Scatchard analysis. The 1G7 binding sites in these cell lines ranged from 160 to 2,200 FcRH5 molecules per cell. Even with very low target copy number, anti-FcRH5/CD3 induced killing of all tested cell lines. Despite the limited maximal killing seen in the case of one cell line, EC_{50} values for all cells were in the pM range (EC_{50} = 2–230 pM, Figure 5C). Occupancy calculations indicate that as few as ~50 TDB molecules (2% occupancy at MOLP-2; EC_{50} = 58 pM) are sufficient to induce T cell activation and target cell apoptosis.

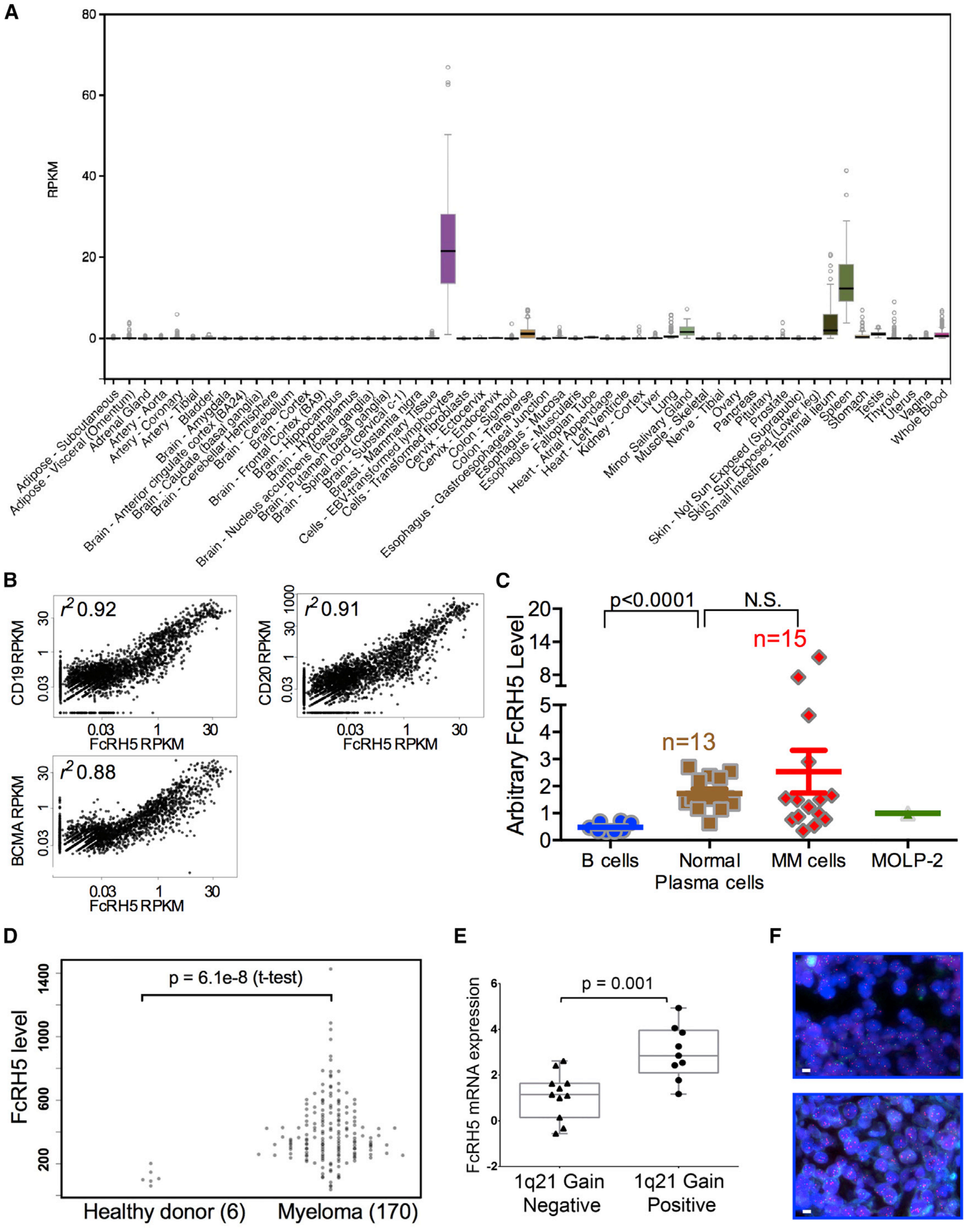

Figure 4. Expression of FcRH5 in Normal Tissues and Multiple Myeloma

(A) FcRH5 RNA-seq analysis of the Genotype-Tissue Expression (GTEx) sample set. Boxes represent the interquartile range (IQR, 25th to 75th percentile), the horizontal line is the median. Whiskers extend to the most extreme data point that is within 1.5*IQR of the 25th and 75th percentiles. Data points beyond the whiskers represent outliers.

(B) Correlation of FcRH5 expression with CD19, CD20, and BCMA expression in the GTEx samples.

(C) FcRH5 protein expression in primary multiple myeloma tumor cells and healthy donor peripheral B cells and bone marrow plasma cells. FcRH5 expression was analyzed by flow cytometry normalized to expression in MOLP-2 internal and assay control. Data are represented as the mean ± SEM.

(D) FcRH5 mRNA expression in anti-CD138 purified plasma cells from newly diagnosed non-treated multiple myeloma patients measured using Affymetrix GeneChip Human Exon 1.0 ST.

(legend continued on next page)

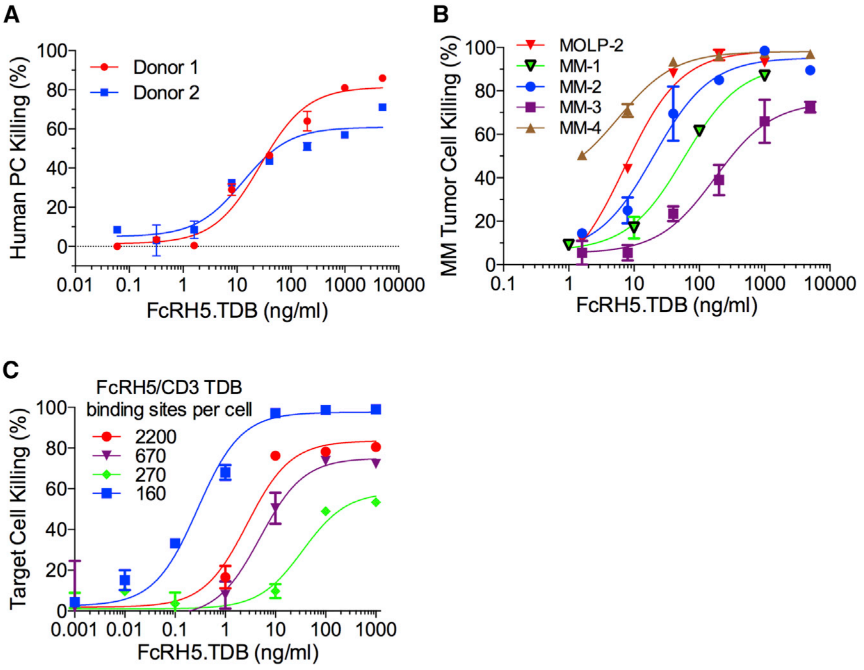

Figure 5. Anti-FcRH5/CD3 TDB Mediates Potent Killing of Normal Plasma Cells and Patient-Derived Primary Myeloma Cells

(A) Cytotoxic activity on human plasma cells was evaluated by culturing human bone marrow mononuclear cells (BMMC) for 72 hr with anti-FcRH5/CD3 TDBs and analyzing the number of live $CD38^+CD138^+$ cells by flow cytometry.

(B) Cytotoxic activity of anti-FcRH5/CD3 TDB on patient-derived primary myeloma cell was evaluated by co-culturing human myeloma BMMCs with $CD8^+$ T cells isolated from healthy donor and anti-FcRH5/CD3 TDB. The killing activity was analyzed as in (A).

(C) The number of anti-FcRH5/CD3 TDB binding sites per cell was determined using Scatchard analysis and is indicated in the panel. Cytotoxic activity of anti-FcRH5/CD3 TDB against target cells expressing low levels of FcRH5 was assessed as in (B). Data are represented as the mean ± SD.

Anti-FcRH5/CD3 TDB Suppresses Growth of Established MOLP-2 Tumors in Mice Reconstituted with Human Immune Cells

Modeling anti-myeloma activity of the anti-FcRH5/CD3 TDB in mice is challenging since anti-CD3 antibodies do not cross-react with mouse CD3 and there is no mouse FcRH5 ortholog. Therefore we established a mouse model with a reconstituted human immune system by transplanting $CD34^+$-selected human hematopoietic stem cells into irradiated mice (humanized NOD/SCID gamma [huNSG] mice). Human $CD8^+$ cells harvested from spleens of huNSG mice were shown to be able to kill MOLP-2 cells in vitro with comparable efficiency as human peripheral $CD8^+$ T cells from healthy donors (Figure 6A). Twenty weeks post transplantation, huNSG mice were inoculated with 20 million MOLP-2 cells subcutaneously. Mice with established tumors (100–200 mm^3) were treated with a weekly intravenous dose of vehicle or 0.5 mg/kg of anti-FcRH5/CD3 TDB. Anti-FcRH5/CD3 TDB treatment resulted in tumor regression in all animals (Figure 6B), indicating that anti-FcRH5/CD3 suppresses tumor growth in vivo.

Cynomolgus Monkey Is an Appropriate Preclinical Model for the Anti-FcRH5/CD3 TDB

Flow cytometry analysis was used to confirm both the reactivity of anti-FcRH5 1G7 to FcRH5 and expression of the target in B cells and plasma cells in cynomolgus monkey (Figures S3A–S3C). FcRH5 expression was detected throughout the B cell lineage in a similar manner to human (Polson et al., 2006), and the anti-FcRH5/CD3 TDB binds to FcRH5 and CD3 with comparable affinity. Treatment with anti-FcRH5/CD3 TDB of target cells expressing primate FcRH5 or MOLP-2 cells expressing human FcRH5 resulted in robust killing using peripheral T cells from either human or cynomolgus monkey with comparable efficiency (Figures S3D and S3E). Adding anti-FcRH5/CD3 TDB to peripheral blood mononuclear cell (PBMC)/BMMC samples from cynomolgus monkey also resulted in a dose-dependent and robust killing of B cells (Figure S3F) and bone marrow plasma cells (Figure S3G). These results validate the cynomolgus monkey as an appropriate safety and efficacy model for the anti-FcRH5/CD3 TDB.

Anti-FcRH5/CD3 TDB Has a Long Serum Half-Life in Cynomolgus Monkeys

A single-dose study was conducted to evaluate efficacy, pharmacokinetic (PK) and pharmacodynamic (PD) properties of the anti-FcRH5/CD3 TDB in cynomolgus monkeys. Monkeys were treated with a single intravenous dose with slow infusion of vehicle or 1, 2, or 4 mg/kg of anti-FcRH5/CD3 TDB. Blood samples were collected for analysis of PK/PD response and cytokines for 7 days after the anti-FcRH5/CD3 TDB was administered. The study was terminated at day 8.

The anti-FcRH5/CD3 TDB demonstrated dose-proportional exposure (C_{max} and area under the curve) between 1 and 4 mg/kg and with clearance ranging from 29 to 33 mL/day/kg in all cohorts (Figure 7A). The C_{max} at the 4 mg/kg dose level was 129 μg/mL, ~2,000-fold higher than that required to reach

(E) qRT-PCR analysis of FcRH5 mRNA level in bone marrow biopsies from patient myeloma samples with or without 1q21 gain. mRNA expression level was calculated by the delta Ct (dCt) method. Statistical analysis was performed using a Mann-Whitney U test. The box represents the 25th to 75th percentiles and median. Whiskers represent the range of minimum and maximum values.

(F) Representative images of FISH analysis on primary multiple myeloma biopsies showing normal diploid of 1q21 (red; top panel) and a mixture of three to six copies of 1q21 (bottom). A tumor sample was identified as 1q21 gain when >20% of the tumor cells scored had three or more copies of the 1q21.3 locus. Scale bars, 10 μm in length.

CellPress

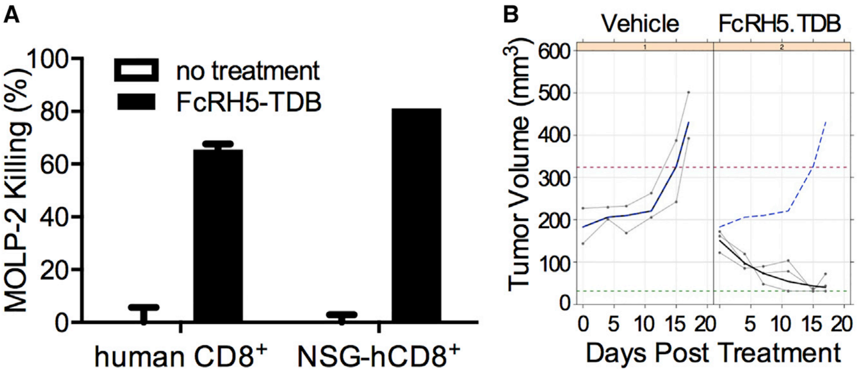

Figure 6. Anti-FcRH5/CD3 TDB Suppresses Growth of Established MOLP-2 Tumors in Mice Reconstituted with Human Immune Cells

(A) MOLP-2 killing activity of peripheral T cells from healthy human donors and of human $CD8^+$ T cells isolated from spleens of humanized NOD/SCID gamma (huNSG) mice. Data are represented as the mean ± SD.

(B) The mice were treated with a weekly intravenous dose of vehicle or anti-FcRH5/CD3 TDB at 0.5 mg/kg. The individual tumor volumes (gray), mean tumor volume (blue bold line in the vehicle-treated group; black bold line in the anti-FcRH5/CD3 TDB-treated group), and mean of control group (blue dashed line) are shown.

the in vitro killing EC_{50} for human plasma cells and MOLP-2. Receptor occupancy calculations suggested near-total saturation of FcRH5 engagement on peripheral blood B cells at C_{max} at all dose levels (Figure S4A). In summary, these results demonstrate that the anti-FcRH5/CD3 TDB has PK characteristics that support an intermittent weekly or less frequent dosing schedule.

Anti-FcRH5/CD3 TDB Depletes B Cells and Bone Marrow Plasma Cells in Cynomolgus Monkeys

Flow cytometry of peripheral blood demonstrated a robust pharmacologic effect at all dose levels. Anti-FcRH5/CD3 treatment resulted in T cell activation and a transient decrease in T cells likely reflecting a margination response within 24 hr (Figures 7B, 7C, S4B, and S4C). $CD4^+$ and $CD8^+$ T cells recovered to baseline levels by the end of the study. In contrast, B cells remained to be undetectable in blood 7 days after anti-FcRH5/CD3 TDB administration indicating that they were depleted by exposure to the molecule (Figure 7D). All dose levels resulted in complete depletion of B cells in spleen and bone marrow (Figures 7E and 7F). Anti-FcRH5/CD3 TDB treatment also induced a robust, dose-dependent depletion of B cells from the lymph nodes (Figures 7G and S4D).

Depletion of primate bone marrow plasma cells is a key efficacy endpoint in the preclinical development of the anti-FcRH5/CD3 TDB. Complete depletion of plasma cells following anti-FcRH5/CD3 TDB treatment was observed in the animals treated at 2 and 4 mg/kg (Figure 7H). Anti-FcRH5/CD3 treatment also resulted in a dose-dependent reduction of IgG of 37% and 44% in the 2 and 4 mg/kg groups, respectively (Figure 7I), an expected secondary outcome resulting from plasma cell depletion. These figures compare favorably with calculations based on the half-life of IgG suggesting that complete depletion of plasma cells would decrease IgG level ~30%–40% by day 7. In summary, anti-FcRH5/CD3 TDB induced a robust PD response in cynomolgus monkeys consistent with its mechanism of action. Complete plasma cell depletion with subsequent decrease in serum IgG levels provides compelling evidence of TDB activity in the bone marrow microenvironment.

Anti-FcRH5/CD3 TDB Induces Transient Cytokine Release in Cynomolgus Monkey

Consistent with the mechanism of action, treatment with the anti-FcRH5/CD3 TDB at all dose levels tested induced a rapid, generally mild/moderate cytokine release (Figure S5), including elevations in IL-6, IL-5, IFN-γ, IL-2, IL-13, and MCP-1 as well as the anti-inflammatory response peaking at 2–6 hr. All cytokines returned to normal baseline levels within 24 hr. In summary, no signs of severe or prolonged cytokine release were observed at dose levels that are expected to saturate target and sufficient for complete depletion of B cells and plasma cells. A single dose at the ≤ 4 mg/kg dose level did not result in detectable neurotoxicity.

PD-L1 Blockade Enhances the Activity of Anti-FcRH5/CD3 TDB

A strong TCR stimulation signal normally leads to immunosuppressive feedback that limits T cell activity. Signaling through the PD-1/PD-L1 axis is a critical component of this feedback and a therapeutically validated immune escape mechanism in several tumor indications. PD-L1 is frequently expressed by myeloma cells (Gorgun et al., 2015), and the signaling axis may limit T cell activity in myeloma patients.

In vitro stimulation (48 hr) of human healthy donor $CD8^+$ cells with anti-FcRH5/CD3 TDB in the presence of target-expressing cells resulted in significant PD-1 induction in T cells (Figure 8A). This feedback signal was also observed in vivo. A significant increase in the number of PD-1-positive T cells was observed when cynomolgus monkeys were treated with anti-FcRH5/CD3 TDB at all tested dose levels. PD-1 induction was detected in both $CD8^+$ and $CD4^+$ T cells in blood, spleen, lymph nodes, and bone marrow (Figures 8B and S6). The ability of anti-FcRH5/CD3 TDB-primed $CD8^+$ T cells to kill PD-L1-expressing target cells was modest (Figure 8C); however, blocking PD-1/PD-L1 signaling using an anti-PD-L1 antibody in combination with anti-FcRH5/CD3 significantly increased the efficiency of killing (Figure 8C). Together, these results show that anti-FcRH5/CD3 TDB-mediated activation of T cells leads to induction of PD-1 in T cells in vitro and in vivo. Although PD-1/PD-L1 signaling can limit anti-FcRH5/CD3 TDB-mediated killing, PD-L1 blockade can overcome this inhibition and lead to enhanced activity of the anti-FcRH5/CD3 TDB. Our data provide strong scientific rationale for the clinical assessment of anti-FcRH5/CD3 TDB combined with anti-PD-L1 blockade in MM patients.

DISCUSSION

Several CD3-bispecific antibodies or antibody fragment-based molecules are in clinical development and clinical

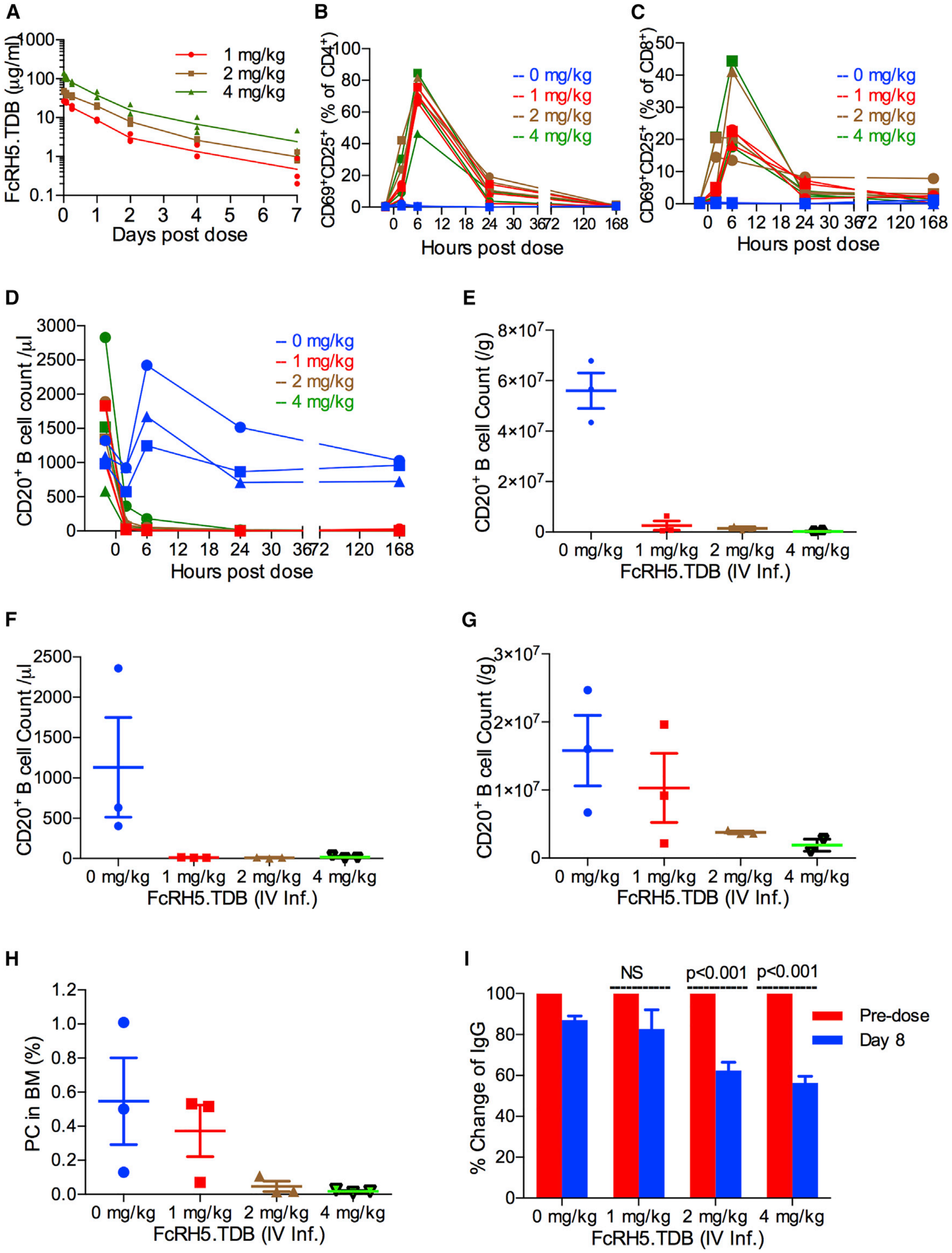

Figure 7. Anti-FcRH5/CD3 TDB Depletes B Cells and Bone Marrow Plasma Cells in Cynomolgus Monkey

A single intravenous dose of vehicle (blue) or anti-FcRH5/CD3 TDB (red, 1 mg/kg; brown, 2 mg/kg; green, 4 mg/kg) was administered to three cynomolgus monkeys/group.

(A) Blood samples were collected at indicated time points and human IgG was detected by ELISA.

(B and C) The effect of anti-FcRH5/CD3 TDB on CD4+ (B) and CD8+ (C) T cell activation in peripheral blood.

(D–G) The absolute count of CD20+ B cells in peripheral blood (D), spleen (E), bone marrow (F), and mandibular lymph node (G).

(H and I) The effect of anti-FcRH5/CD3 TDB on bone marrow plasma cells (H) and serum IgG (I) levels. The difference between before and after treatment was analyzed by unpaired t test. Data in E–I are represented as the mean ± SEM. See also Figures S3–S5.

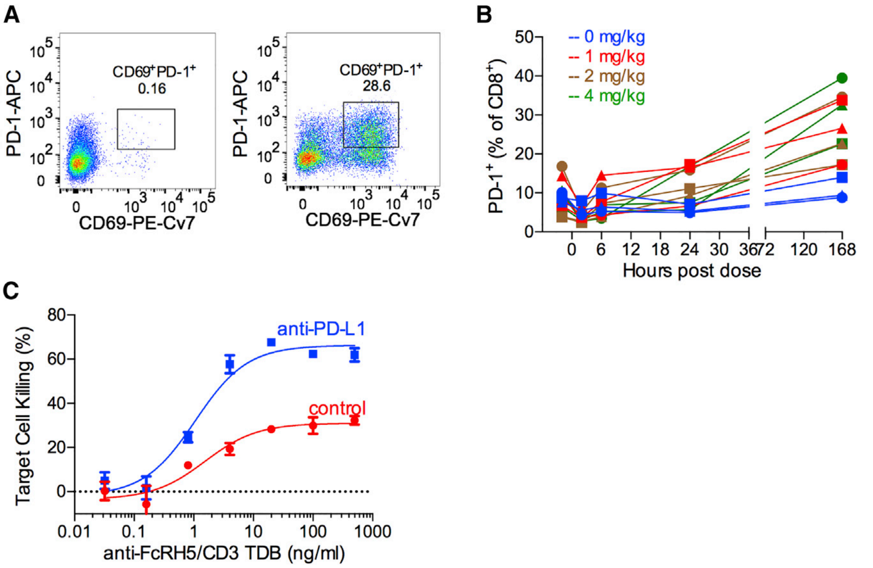

Figure 8. PD-L1 Blockade Enhances Activity of Anti-FcRH5/CD3 TDB

(A) $CD8^+$ T cells were stimulated for 48 hr with 1 μg/mL anti-FcRH5/CD3 TDB and MOLP-2 target cells, and analyzed by flow cytometry for the PD-1 expression.

(B) The percentage of $PD-1^+$ cells in $CD8^+$ T cells in cynomolgus monkeys after single-dose intravenous administration of vehicle (blue), 1 mg/kg (red), 2 mg/kg (brown), and 4 mg/kg (green) of anti-FcRH5/CD3 TDB. Individual animal data are shown.

(C) The ability of anti-FcRH5/CD3 TDB to redirect activity of pre-stimulated $CD8^+$ T cells (A) to kill 293-FcRH5-PD-L1 cells was tested in the presence (blue) or absence (red) of 10 μg/mL anti-PD-L1. Data are represented as the mean ± SEM. See also Figure S6.

proof-of-concept has been established by blinatumomab in hematological malignancies (Bargou et al., 2008). Optimally, the CD3-bispecific molecules show extremely potent preclinical activity regardless of the target molecule or the molecule format. In our studies, using a plethora of tumor targets and antibody clones, we have detected extreme variability in the activity of the molecules that could not explained by the affinities of the molecules. In this study we describe the molecular events in the synapse induced by a CD3-bispecific full-length antibody and show that the events closely follow the principles of kinetic segregation, and are not significantly different compared with TCR activation by pMHC ligation. Our studies confirm that FcRH5 is suboptimal for bispecific antibody-mediated T cell triggering due to its large ECD that interferes with efficient synapse formation. By using antibodies that bind to various different epitopes we show that the efficiency of synapse formation correlates with the proximity of binding epitope to cell membrane. The difference between the epitopes in the end result (killing of myeloma cells) is dramatic: membrane-proximal antibodies kill with picomolar concentrations, whereas more distal antibodies are essentially inert. Potency in the context of BiTE-mediated killing has been correlated with similar structural features of the target molecule (Bluemel et al., 2010) complementing our molecular studies of synapse and mechanism of T cell triggering.

Anti-FcRH5/CD3 TDB killed patient-derived myeloma cells and healthy donor-derived plasma cells at picomolar concentrations. Non-clinical pharmacology studies with anti-FcRH5/CD3 TDB in mice are challenging. First, anti-FcRH5/CD3 TDB is not reactive to mouse CD3. Second, an FcRH5 ortholog does not exist in the mouse; thus genetically engineered mouse models are not suitable for testing the molecule. The only available mouse tumor model that can be used is human myeloma cell line (MOLP-2) xenografted to immune-compromised mice supplemented with human T cells. As these tumors are grafted subcutaneously, the MOLP-2 xenografts do not model activity in the bone marrow environment. A further clear limitation of this type of xenograft model is that the immune system engrafted in the mice likely does not exactly recapitulate the adult human immune system, potentially not accounting for the contribution of other cell types, such as T_{reg} cells, to TDB treatment. In contrast, cynomolgus monkeys can be used as a compelling efficacy model to demonstrate in vivo activity in the bone marrow compartment. A single dose of the anti-FcRH5/CD3 TDB depleted plasma cells and B cells from tissues and led to expected reduction of serum IgG levels.

Cytokine release has been reported consistently across CD3-targeting bispecific molecule platforms with variable frequency and severity. Clinical cytokine release syndrome (CRS) has been reported in CD19-targeting agents, e.g., blinatumomab and CD19 CAR-T cells. As expected, the anti-FcRH5/CD3 TDB induced mild/moderate cytokine release immediately after dose administration, but no extensive or prolonged cytokine release was observed in cynomolgus monkeys. The predictive value of the detected cytokine levels in primates to CRS in myeloma patients is unclear. However, several potential mitigation strategies are available for cytokine-related adverse effects (dose fractionation, corticosteroids, or IL-6 signaling blockers).

The anti-FcRH5/CD3 TDB is predicted to be broadly active in myeloma as the prevalence of the target expression is 100%, and as few as ~200 copies of FcRH5 on a MM cell are sufficient to induce tumor cell killing. In addition to myeloma, evidence of frequent FcRH5 expression has been reported in multiple B cell malignancies such as chronic lymphocytic leukemia, mantle cell lymphoma, diffuse large B cell lymphoma, and follicular lymphoma (Ise et al., 2007; Li et al., 2008; Polson et al., 2006). This suggests a more general applicability for anti-FcRH5/CD3 in B cell-mediated malignancies in addition to MM.

Gain or amplification of chromosome 1q21 is one of the most commonly detected genetic abnormalities in MM and is considered a predictive marker of aggressive disease (Boyd et al., 2012). FcRH5 was originally identified in cloning of this chromosomal region and has been shown to be deregulated in cell lines with 1q21 abnormalities (Hatzivassiliou et al., 2001). Our analysis of primary myeloma samples demonstrates that FcRH5 mRNA is elevated in myeloma patients with 1q21 gain. Although it is unlikely that FcRH5 plays a functional role in the myeloma

progression, its overexpression provides an intriguing diagnostic hypothesis for the anti-FcRH5/CD3 TDB. Our in vitro assays demonstrate a correlation between target expression level and activity of the molecule, suggesting that MM with gain of 1q21 may be exquisitely sensitive to the anti-FcRH5/CD3 TDB, thus providing clinical benefit to a patient population that otherwise has very limited treatment options.

T cell activation by anti-FcRH5/CD3 TDB induced upregulation of PD-1 in T cells. This negative feedback signaling has been detected with other CD3 targeting bispecific molecules regardless of the format of the molecule (Bacac et al., 2016; Junttila et al., 2014; Osada et al., 2015), and is likely a class effect for molecules with this mechanism of action. As PD-1/PD-L1 signaling can inhibit the killing activity of T cells, optimal clinical use of T cell-recruiting bispecific antibodies may include combination with inhibitors for this pathway.

EXPERIMENTAL PROCEDURES

Antibodies

Antibodies were from BD Biosciences unless otherwise mentioned. Anti-human PD-1 was from Affymetrix. Goat anti-human IgG and goat anti-mouse IgG were from Jackson ImmunoResearch. Anti-PC-FITC (clone Vs38c) was from Dako. SLP-76 from Cell Signaling Technology. p-SLP76 (Ser376) and anti-PD-L1 antibodies were generated at Genentech.

Fluorescent Labeling of Antibodies

For detection of FcRH5 from MM samples and healthy donor plasma and B cells by flow cytometry, anti-FcRH5 antibody 1G7 was labeled with phycoerythrin by SouthernBiotech. For microscopy, the TDBs were labeled with Alexa Fluor 647 using the appropriate protein labeling kit (Thermo Fisher Scientific) according to the manufacturer's instructions. TDBs were dialyzed into PBS, pH 7.2, prior to labeling and a dye/protein ratio of ~4 was routinely achieved.

Stable Cell Lines

To evaluate the immunological synapse formation, SVT2 cells were infected with retrovirus encoding full-length FcRH5 with N-terminal gD expression tag or truncated FcRH5 (deletion of AA1-744) with N-terminal gD tag. To evaluate the target dependency of TDB killing, FOX-NY cells were infected with lentivirus encoding full-length FcRH5 and single-cell-derived clones with differential expression level of FcRH5 were selected. To evaluate the effect of PD-1/PD-L1 signaling to TDB activity, 293 cells were infected with lentivirus encoding FcRH5 followed by transfection of human PD-L1 encoding plasmid using Lipofectamine (Invitrogen).

Vectors and Transient Transfection for Microscopy

FcRH5 with N-terminal gD expression tag was fused to the fluorescent protein mRuby2 by first inserting FcRH5 into the pHR-SIN lentiviral vector, before ligating the mRuby2 DNA sequence into this vector, creating pHR-FcRH5-mRuby2. The SFFV promoter in this vector was subsequently replaced with the mHSP promoter, creating pHRI-FcRH5-mRuby2, which utilizes a weaker promoter than pHR, allowing more physiological expression levels of FcRH5-Ruby. Vectors expressing LCK, ZAP70, CSK/CBP, and CD45 have been described previously (James and Vale, 2012). The CD45 construct used was either the RO isoform or a construct containing the cytoplasmic domain of CD45 with the transmembrane and ECDs of CD43, which is known to mimic the function of CD45. Vectors were transiently transfected at appropriate ratios using GeneJuice (Novagen), Cells were used in experiments 24–48 hr after transfection.

Microscopy Imaging and Analysis

To image cell conjugates, 3×10^5 cells were harvested from culture and resuspended in 100 μL of 20 nM TDB in RPMI-1640 (without phenol red). After 20–30 min incubation to allow cell conjugation, cells were washed with PBS, resuspended in DMEM^{gfp2} imaging medium (Evrogen) and added to 35 mm imaging dishes (Mattek). An Andor spinning disc confocal microscope system was used to image the cells at 37°C. All images were analyzed and all presented images were manipulated in an equivalent manner using ImageJ. The presented images were background subtracted and then cropped to focus on the pair of cells and the contrast was optimized. The degree of protein clustering and segregation was determined by using the intensity of fluorescently labeled proteins in the plasma membrane. The plasma membrane was selected by manually drawing a line and the average fluorescence intensity of the plasma membrane within the cell-cell interface was divided by the average fluorescence intensity of the plasma membrane outside the cell-cell interface to calculate the degree of clustering or segregation. To generate an image of the interface of a pair of cells conjugated by TDBs from a z stack, the image stack was first deconvolved and then cropped to highlight the interface region using Huygens software.

Production of TDBs

Full-length bispecific antibodies were produced as described elsewhere (Junttila et al., 2014; Sun et al., 2015). In brief, the two half antibodies containing the "knob" or the "hole" mutations in the CH3 domains were expressed by transient transfection of CHO cells and then affinity purified with Protein A. Equal amounts of the two half antibodies were incubated with a 200 molar excess of reduced glutathione at pH 8.5 overnight at 32°C to drive the formation of the knob-hole disulfide bonds. The assembled bispecific antibody was purified from contaminants through hydrophobic interaction chromatography.

In Vitro Cytotoxicity Assays: Cell Lines

PBMCs and CD8$^+$ separation, CellTiter-Glo (Promega) and flow cytometry-based viability assays (48 hr) were described previously (Junttila et al., 2014). CD4$^+$ T cells were isolated from PBMCs by the Human CD4$^+$ T cell Isolation Kit (Miltenyi Biotec). CD4$^+$ or CD8$^+$ cells were used as effectors in a 3:1 effector:target ratio.

In Vitro Cytotoxicity Assay: Human Plasma Cells and Primary MM Samples

Human BMMCs from MM patients were procured from Conversant Bio. Human bone marrow aspirates of healthy donors were procured from AllCells. All human biospecimens were collected, processed, and distributed in full ethical and regulatory compliance with the sites from which human biospecimens were collected. This includes independent ethical review, institutional review board approval (where appropriate), independent regulatory review, and ethical review for collection sites. All sites were located in the US and the EU. In vitro experiments using human healthy donor blood, bone marrow, or vendor-procured live tumor material are routinely performed at Genentech and do not require approval by an internal ethical review committee.

Human bone marrow aspirates of healthy donors were diluted in PBS and BMMCs were isolated by conventional gradient separation (Lymphoprep, STEMCELL). Flow cytometry viability assay was used to test the effect of 72 hr anti-FcRH5/CD3 TDB treatment on BMMC plasma cells. Myeloma BMMCs were mixed with freshly isolated healthy donor CD8$^+$ T cells and co-culture treated with anti-FcRH5/CD3 TDB for 72 hr. PI-negative CD38$^+$CD138$^+$ cells were counted by flow cytometry. The killing activity was calculated as: {(number of live target cells without TDB − number of live target cells with TDB)/(number of live target cells without TDB)} × 100%.

T Cell Activation

Human CD4$^+$ T cells, CD8$^+$ T cells, or T_{reg} cells (CD4$^+$CD25$^+$CD127^{low}) were mixed with MOLP-2 cells in a 3:1 ratio and co-cultured with Anti-FcRH5/CD3 TDB for 24 hr. The T cell activation assay has been described previously (Junttila et al., 2014).

T Cell Proliferation

CD8$^+$ T cells were labeled with carboxyfluorescein succinimidyl ester and co-cultured with MOLP-2 cells (1:1) and 1 μg/mL TDB for 5 days.

Western Blot Analysis

pSLP76 (Ser376) and SLP76 were analyzed in human CD8$^+$ T cells co-cultured with HEK293-FcRH5 cells (2:1) and 1 μg/mL of TDBs.

RNA Expression in Normal Tissues and Myeloma Samples

mRNA expression was analyzed in the GTEx RNA sequencing sample set (Ardlie et al., 2015) consisting of 8,555 samples from 544 donors over 53 tissues and in the NCBI GEO: GSE39754 dataset (Affymetrix GeneChip Human Exon 1.0 ST Array) from the NCBI GEO repository (Chauhan et al., 2012). CD138 purified plasma cell samples in the NCBI GEO: GSE39754 dataset represent newly diagnosed patients with MM before initiation of primary treatment.

RNA Isolation, cDNA Synthesis, and Gene Expression Analysis

Total RNA was extracted from decalcified formalin-fixed paraffin-embedded (FFPE) bone marrow biopsy tissues collected from MM patients. Two reference genes, SDHA and VPS33B, were evaluated for each sample and used to calculate expression of FcRH5. Gene expression of FcRH5 was determined by using the delta Ct (dCt) method ($Ct_{gene\ of\ interest} - Ct_{geometric\ mean\ of\ reference\ genes}$). Detailed description of method in the supplement.

Cytogenetic Fluorescent In Situ Hybridization

1q21 + copy control 1 fluorescent in situ hybridization (FISH) probe (Biocare Medical; previously CymoGen Dx) was used to analyze the 1q21 region. The 1q21 probe covers the chromosomal band 1q21.3 while the control probe is located in the peri-centromeric 1p12 region of chromosome 1. FISH analysis on FFPE tissue was performed as described previously (Koeppen et al., 2014; O'Brien et al., 2008). A minimum of 100 non-overlapping tumor cells from each sample was enumerated. Cutoff of gain was 3 or more copies in >20% of the tumor cells. Detailed description of method in the supplement.

huNSG/MOLP-2 Mouse Xenograft Model

All mouse experimental procedures conformed to the guiding principles of the American Physiology Society and were approved by Genentech's Institutional Animal Care and Use Committee (IACUC). Female huNSG mice were obtained from The Jackson Laboratory. Animals were inoculated with 20 million MOLP-2 tumor cells in Hank's balanced salt solution/Matrigel, subcutaneously. Treatments were administered intravenously once a week ×4. Detailed description of method in the supplement.

Cynomolgus Monkey Study

The PK and PD properties of anti-FcRH5/CD3 TDB were evaluated in naive, male cynomolgus monkeys (cynos) at Charles River Laboratories (CRL). Cynos were treated with a single-dose, intravenous infusion (1 hr) of vehicle, 1, 2, or 4 mg/kg anti-FcRH5/anti-CD3 TDB blood samples were collected by venipuncture via the femoral vein pre-study and at selected time points for 7 days after dosing for analyses of hematology, serum chemistry, coagulation, and PK and PD endpoints (cytokines, flow cytometry of T lymphocytes, B lymphocytes, activated T lymphocytes, and PD-1 and circulating cyno IgG). Bone marrow was collected in anesthetized animals by aspiration from the humerus pre-study and on day 8 for evaluation of B lymphocytes and plasma cells by flow cytometry. The study was terminated at day 8. All procedures were approved by the CRL IACUC and were performed in compliance with the Animal Welfare Act, the Guide for the Care and Use of Laboratory Animals, and the Office of Laboratory Animal welfare.

Anti-FcRH5/CD3 Pharmacokinetics in Cyno

Anti-FcRH5/CD3 TDB in serum were determined by generic ELISA. Sheep anti-human IgG antibody was used as the capturing reagent and sheep anti-human IgG conjugated to horseradish peroxidase (HRP) was used as the detection reagent. Serum concentration-time data from available samples were analyzed by a non-compartmental with IV bolus input model (Phoenix WinNonlin, Version 6.3; Pharsight Corporation). Nominal sample collection time and nominal dose concentrations were used in the data analysis. All TK analyses were based on individual animal data.

Flow Cytometry Analysis for Cyno Plasma Cells

Cyno bone marrow aspirate were diluted (1:10) into ammonium-chloride-potassium lysis buffer twice. Cyno bone marrow cells were stained with anti-CD45, anti-CD20, and anti-CD38. After wash, cells were fixed and permeabilized with IntraStain Kit (Dako). Cells then were stained with anti-PC (clone Vs38c). The cyno plasma cells were classified by flow cytometry as $CD45^{-}CD20^{-}CD38^{+}PC^{+}$.

ELISA Analysis for Cyno IgG Level

Total cyno serum IgG was quantified using standard colorimetric-based sandwiched ELISA. A goat anti-monkey IgG (Bethyl Laboratories, A140-202A) and a HRP conjugated goat anti-monkey IgG (Bethyl Laboratories, A140-202P) were used as the capture and detection antibody, respectively. Cyno IgG (Cell Sciences CSI20163A) was used as the protein quantification standard.

PD-1 Induction and Cytotoxicity Assay with anti-PD-L1

Human $CD8^{+}$ T cells and MOLP-2 cells were co-cultured (1:1) with 1,000 ng/mL of TDB for 48 hr and stained with anti-PD-1-APC. Cytotoxicity assay is described above.

SUPPLEMENTAL INFORMATION

Supplemental Information includes Supplemental Experimental Procedures and six figures and can be found with this article online at http://dx.doi.org/10.1016/j.ccell.2017.02.001.

AUTHOR CONTRIBUTIONS

Conceptualization, J.L., J.R.J., and T.T.J.; Methodology, J.L., N.S., J.J., M.J.H., S.A.M., D.D.V.C., M.H., R.C., D.S., R.N., L.M., S.S., E.L., Z.Y., T.D.W., T.S., D.E., I.H., J.R.J., and T.T.J.; Investigation, J.L., N.S., J.J., M.J.H., S.A.M., D.D.V.C., M.H., R.C., D.S., R.N., L.M., S.S., E.L., Z.Y., T.D.W., T.S., D.D., K.T., I.H., D.E., I.H., J.R.J., and T.T.J.; Writing, J.L., J.R.J., and T.T.J.; Supervision, N.S., S.S., Z.Y., K.T., I.H., J.R.J., and T.T.J.; Project Administration, G.Y.L.

ACKNOWLEDGMENTS

This work was supported by a Sir Henry Dale Fellowship (J.R.J.) jointly funded by the Wellcome Trust and the Royal Society (grant number: 099966/Z/12/Z). PhD studentships (S.A.M. and M.J.H.) were funded by the Wellcome Trust (grant number: 102195/Z/13/Z).

Received: August 10, 2016
Revised: November 23, 2016
Accepted: January 31, 2017
Published: March 2, 2017

REFERENCES

Ardlie, K.G., DeLuca, D.S., Segre, A.V., Sullivan, T.J., Young, T.R., Gelfand, E.T., Trowbridge, C.A., Maller, J.B., Tukiainen, T., Lek, M., et al. (2015). The Genotype-Tissue Expression (GTEx) pilot analysis: multitissue gene regulation in humans. Science *348*, 648–660.

Bacac, M., Fauti, T., Sam, J., Colombetti, S., Weinzierl, T., Ouaret, D., Bodmer, W.F., Lehmann, S., Hofer, T., Hosse, R.J., et al. (2016). A novel carcinoembryonic antigen T cell bispecific antibody (Cea Tcb) for the treatment of solid tumors. Clin. Cancer Res. *22*, 3286–3297.

Bargou, R., Leo, E., Zugmaier, G., Klinger, M., Goebeler, M., Knop, S., Noppeney, R., Viardot, A., Hess, G., Schuler, M., et al. (2008). Tumor regression in cancer patients by very low doses of a T cell-engaging antibody. Science *321*, 974–977.

Bluemel, C., Hausmann, S., Fluhr, P., Sriskandarajah, M., Stallcup, W.B., Baeuerle, P.A., and Kufer, P. (2010). Epitope distance to the target cell membrane and antigen size determine the potency of T cell-mediated lysis by BiTE antibodies specific for a large melanoma surface antigen. Cancer Immunol. Immunother. *59*, 1197–1209.

Boyd, K.D., Ross, F.M., Chiecchio, L., Dagrada, G.P., Konn, Z.J., Tapper, W.J., Walker, B.A., Wardell, C.P., Gregory, W.M., Szubert, A.J., et al. (2012). A novel prognostic model in myeloma based on co-segregating adverse FISH lesions and the ISS: analysis of patients treated in the MRC Myeloma IX trial. Leukemia *26*, 349–355.

Chauhan, D., Tian, Z., Nicholson, B., Kumar, K.G.S., Zhou, B., Carrasco, R., McDermott, J.L., Leach, C.A., Fulcinniti, M., Kodrasov, M.P., et al. (2012). A small molecule inhibitor of ubiquitin-specific Protease-7 induces apoptosis

in multiple myeloma cells and overcomes bortezomib resistance. Cancer Cell *22*, 345–358.

Davis, S.J., and van der Merwe, P.A. (2006). The kinetic-segregation model: TCR triggering and beyond. Nat. Immunol. *7*, 803–809.

Elkins, K., Zheng, B., Go, M., Slaga, D., Du, C., Scales, S.J., Yu, S.F., McBride, J., de Tute, R., Rawstron, A., et al. (2012). FcRL5 as a target of antibody-drug conjugates for the treatment of multiple myeloma. Mol. Cancer Ther. *11*, 2222–2232.

Garfall, A.L., Maus, M.V., Hwang, W.T., Lacey, S.F., Mahnke, Y.D., Melenhorst, J.J., Zheng, Z., Vogl, D.T., Cohen, A.D., Weiss, B.M., et al. (2015). Chimeric antigen receptor T cells against CD19 for multiple myeloma. N. Engl. J. Med. *373*, 1040–1047.

Gorgun, G., Samur, M.K., Cowens, K.B., Paula, S., Bianchi, G., Anderson, J.E., White, R.E., Singh, A., Ohguchi, H., Suzuki, R., et al. (2015). Lenalidomide enhances immune checkpoint blockade-induced immune response in multiple myeloma. Clin. Cancer Res. *21*, 4607–4618.

Hatzivassiliou, G., Miller, I., Takizawa, J., Palanisamy, N., Rao, P.H., Iida, S., Tagawa, S., Taniwaki, M., Russo, J., Neri, A., et al. (2001). IRTA1 and IRTA2, novel immunoglobulin superfamily receptors expressed in B cells and involved in chromosome 1q21 abnormalities in B cell malignancy. Immunity *14*, 277–289.

Ise, T., Nagata, S., Kreitman, R.J., Wilson, W.H., Wayne, A.S., Stetler-Stevenson, M., Bishop, M.R., Scheinberg, D.A., Rassenti, L., Kipps, T.J., et al. (2007). Elevation of soluble CD307 (IRTA2/FcRH5) protein in the blood and expression on malignant cells of patients with multiple myeloma, chronic lymphocytic leukemia, and mantle cell lymphoma. Leukemia *21*, 169–174.

James, J.R., and Vale, R.D. (2012). Biophysical mechanism of T-cell receptor triggering in a reconstituted system. Nature *487*, 64–69.

Junttila, T.T., Li, J., Johnston, J., Hristopoulos, M., Clark, R., Ellerman, D., Wang, B.E., Li, Y., Mathieu, M., Li, G., et al. (2014). Antitumor efficacy of a bispecific antibody that targets HER2 and activates T cells. Cancer Res. *74*, 5561–5571.

Koeppen, H., Yu, W., Zha, J., Pandita, A., Penuel, E., Rangell, L., Raja, R., Mohan, S., Patel, R., Desai, R., et al. (2014). Biomarker analyses from a placebo-controlled phase II study evaluating Erlotinib +/− Onartuzumab in advanced non-small cell lung cancer: MET expression levels are predictive of patient benefit. Clin. Cancer Res. *20*, 4488–4498.

Li, F.J., Ding, S., Pan, J., Shakhmatov, M.A., Kashentseva, E., Wu, J., Li, Y., Soong, S.J., Chiorazzi, N., and Davis, R.S. (2008). FCRL2 expression predicts IGHV mutation status and clinical progression in chronic lymphocytic leukemia. Blood *112*, 179–187.

O'Brien, C., Cavet, G., Pandita, A., Hu, X.L., Haydu, L., Mohan, S., Toy, K., Rivers, C.S., Modrusan, Z., Amler, L.C., and Lackner, M.R. (2008). Functional genomics identifies ABCC3 as a mediator of taxane resistance in HER2-amplified breast cancer. Cancer Res. *68*, 5380–5389.

Offner, S., Hofmeister, R., Romaniuk, A., Kufer, P., and Baeuerle, P.A. (2006). Induction of regular cytolytic T cell synapses by bispecific single-chain antibody constructs on MHC class I-negative tumor cells. Mol. Immunol. *43*, 763–771.

Osada, T., Patel, S.P., Hammond, S.A., Osada, K., Morse, M.A., and Lyerly, H.K. (2015). CEA/CD3-bispecific T cell-engaging (BiTE) antibody-mediated T lymphocyte cytotoxicity maximized by inhibition of both PD1 and PD-L1. Cancer Immunol. Immunother. *64*, 677–688.

Polson, A.G., Zheng, B., Elkins, K., Chang, W., Du, C., Dowd, P., Yen, L., Tan, C., Hongo, J.A., Koeppen, H., and Ebens, A. (2006). Expression pattern of the human FcRH/IRTA receptors in normal tissue and in B-chronic lymphocytic leukemia. Int. Immunol. *18*, 1363–1373.

Rapoport, A.P., Stadtmauer, E.A., Binder-Scholl, G.K., Goloubeva, O., Vogl, D.T., Lacey, S.F., Badros, A.Z., Garfall, A., Weiss, B., Finklestein, J., et al. (2015). NY-ESO-1-specific TCR-engineered T cells mediate sustained antigen-specific antitumor effects in myeloma. Nat. Med. *21*, 914–921.

Sadelain, M. (2015). CAR therapy: the CD19 paradigm. J. Clin. Invest. *125*, 3392–3400.

Sun, L.L., Ellerman, D., Mathieu, M., Hristopoulos, M., Chen, X., Li, Y., Yan, X., Clark, R., Reyes, A., Stefanich, E., et al. (2015). Anti-CD20/CD3 T cell-dependent bispecific antibody for the treatment of B cell malignancies. Sci. Transl. Med. *7*, 287ra270.

Cancer Cell, Volume *31*

Supplemental Information

Membrane-Proximal Epitope Facilitates Efficient T Cell Synapse Formation by Anti-FcRH5/CD3 and Is a Requirement for Myeloma Cell Killing

Ji Li, Nicola J. Stagg, Jennifer Johnston, Michael J. Harris, Sam A. Menzies, Danielle DiCara, Vanessa Clark, Maria Hristopoulos, Ryan Cook, Dionysos Slaga, Rin Nakamura, Luke McCarty, Siddharth Sukumaran, Elizabeth Luis, Zhengmao Ye, Thomas D. Wu, Teiko Sumiyoshi, Dimitry Danilenko, Genee Y. Lee, Klara Totpal, Diego Ellerman, Isidro Hötzel, John R. James, and Teemu T. Junttila

Supplemental Data

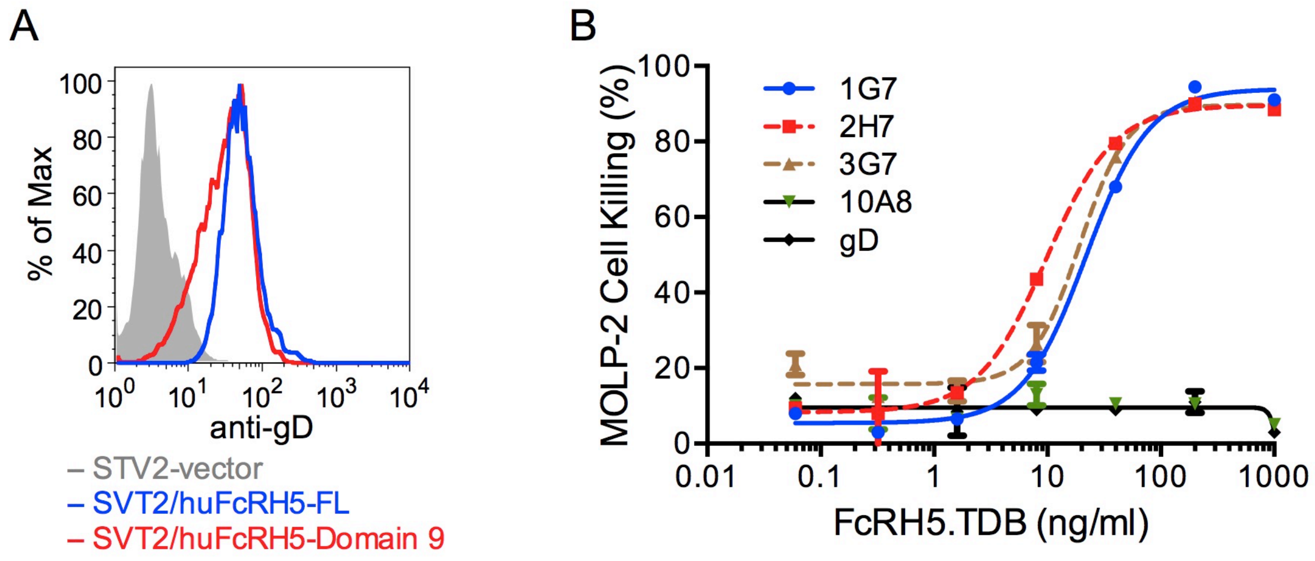

Figure S1. Expression level of full length FcRH5 or truncated FcRH5 in SVT2 cell lines and in vitro activity of multiple TDBs targeting membrane proximal region of the FcRH5. Related to Figure 2. (A) Histogram overlay of flow cytometry of SVT2-parental cells (grey), gD-FcRH5 full length (blue) and gD-FcRH5-domain 9 (red). (**B)** Activity of multiple TDBs targeting FcRH5 on MOLP2 cells. 10A8-TDB binds to distal regions of the endogenously expressed FcRH5. 1G7, 2H7 and 3G7-TDBs all bind to the membrane proximal region of the FcRH5. Data are represented as the mean ± SD.

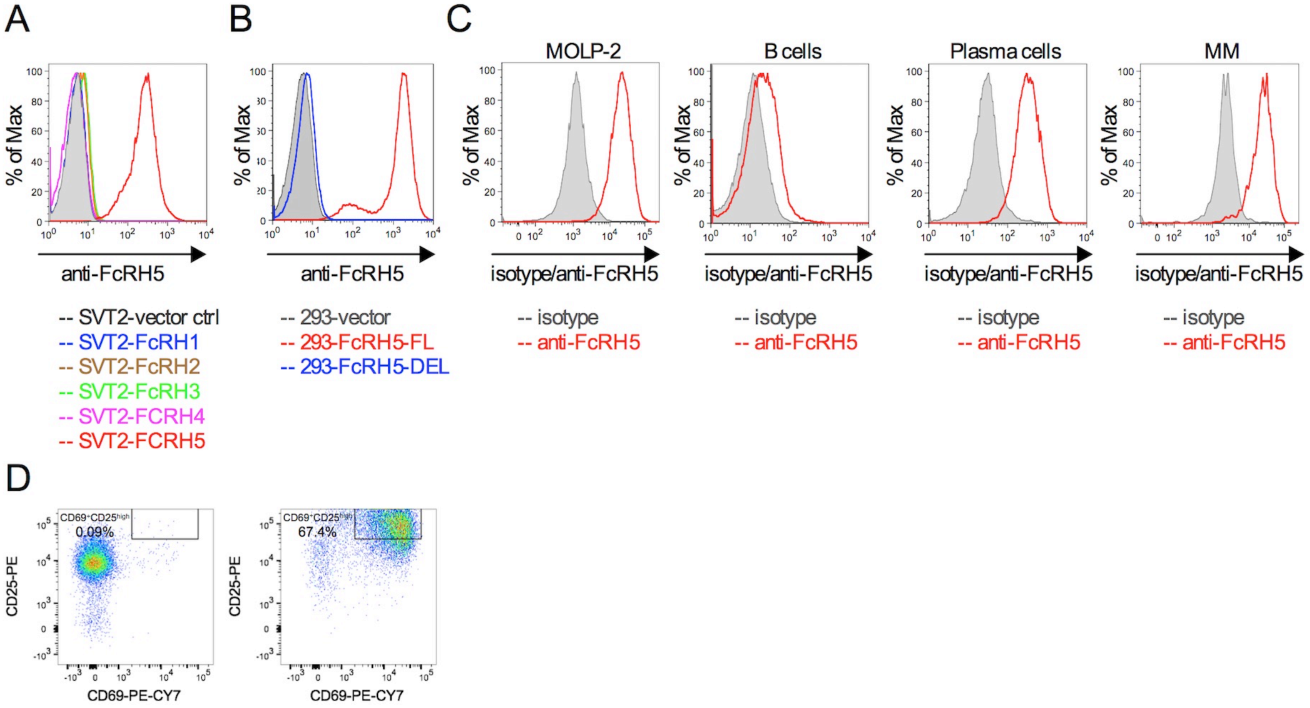

Figure S2. Binding specificity of anti-FcRH5 antibody clone 1G7 and activation of T reg cells by anti-FcRH5/CD3 TDB. Related to Figure 3. (A) Binding of clone 1G7 to FcRH family members. Histogram overlay of 6 cell lines, SVT2-vector (grey), SVT2-FcRH1 (blue), SVT2-FCRH2 (brown), SVT2-FcRH3 (green), SVT2-FcRH4 (purple), and SVT2-FcRH5 (red). **(B)** Histogram overlay of 1G7 binding to 3 cell lines, 293 parental (grey), 293-FcRH5 FL (full length; red) and 293-FcRH5-DEL (Deletion of AA464-850; blue). **(C)** Binding of clone 1G7 to MM cell line and primary cells. Overlay histogram of isotype-PE (grey) and anti-FcRH5 clone 1G7-PE (red) on MOLP-2 cell line, human $CD20^+$ B cells, human $CD38^+CD138^+$ plasma cells and $CD38^+CD138^+$ MM tumor cells from MM bone marrow aspirate. **(D)** Flow cytometry dot plots of T reg activation after 48 hr co-culture of sorted T regs and MOLP-2 in the absence of FcRH5/CD3 TDB (left) or in the presence of 1000 ng/ml of FcRH5/CD3 TDB (right). Activated T regs were classified as $CD69^+CD25^{high}$.

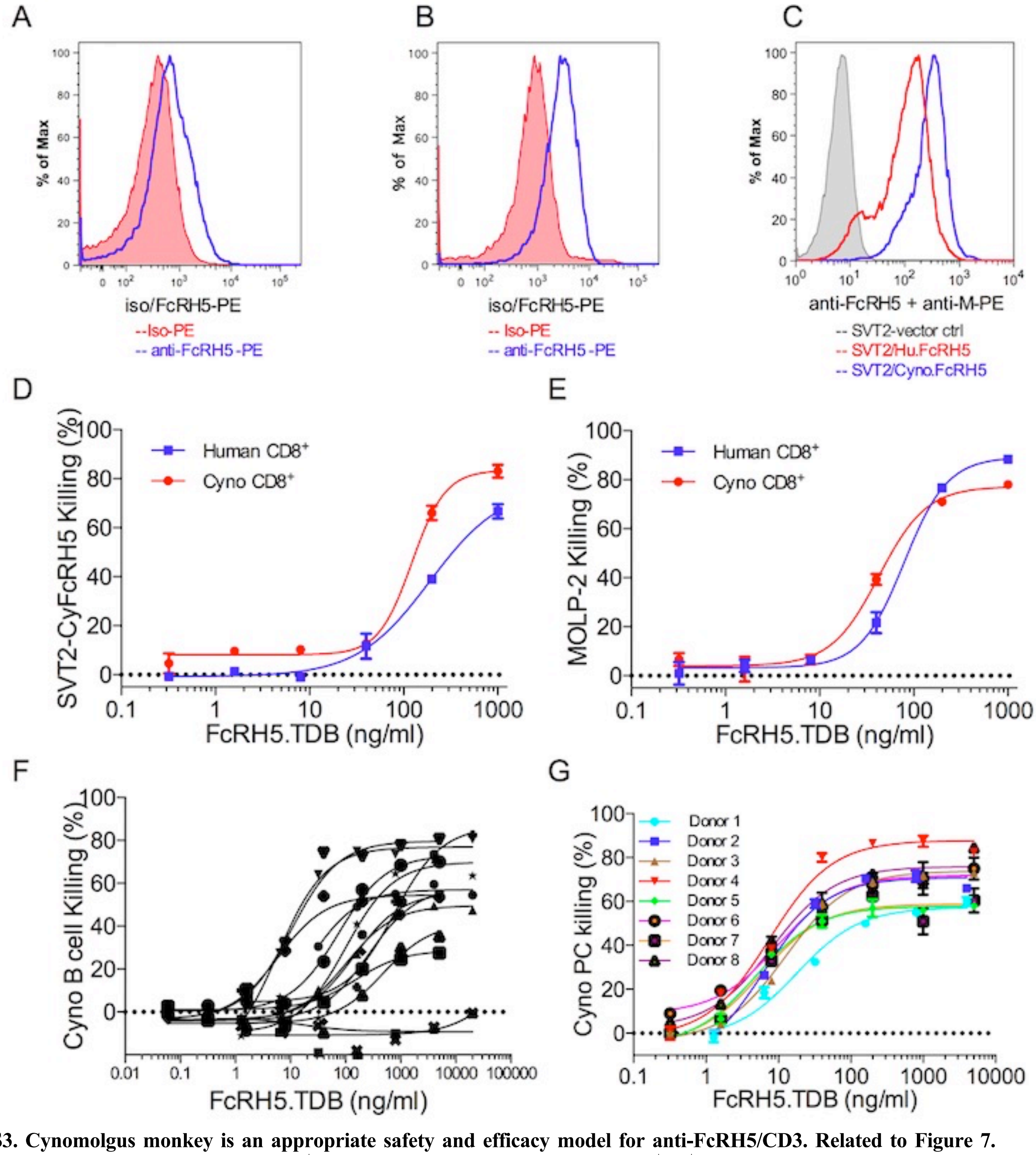

Figure S3. Cynomolgus monkey is an appropriate safety and efficacy model for anti-FcRH5/CD3. Related to Figure 7. TDB. Expression of FcRH5 on cyno CD20^{+} B cells **(A)** and on CD45^{-}CD20^{-}CD38^{+}PC^{+} cyno plasma cells **(B)**. Histogram overlays of isotype-PE (red) and anti-FcRH5 clone 1G7-PE (blue). **(C)** Binding of clone 1G7 to cyno FcRH5. Overlay histogram of 3 cell lines, SVT2-vector (grey), SVT2-hu.FcRH5 (red) and SVT2-cyno.FcRH5 (blue). **(D, E)** Cytotoxic activity of cyno CD8^{+} T cells compared to human CD8^{+} T cells. In vitro killing dose response on SVT2-cyno-FcRH5 **(D)** and MOLP-2 **(E)** with human CD8^{+} T cells (blue) or cyno CD8^{+} T cells (red). **(F)** In vitro killing activity of Anti-FcRH5/CD3 on cyno CD20^{+} B cells (n=14). **(G)** In vitro killing activity of anti-FcRH5/CD3 on CD45^{-}CD20^{-}CD38^{+}PC^{+} plasma cells from cyno bone marrow (n=8). Data are represented as the mean ± SD.

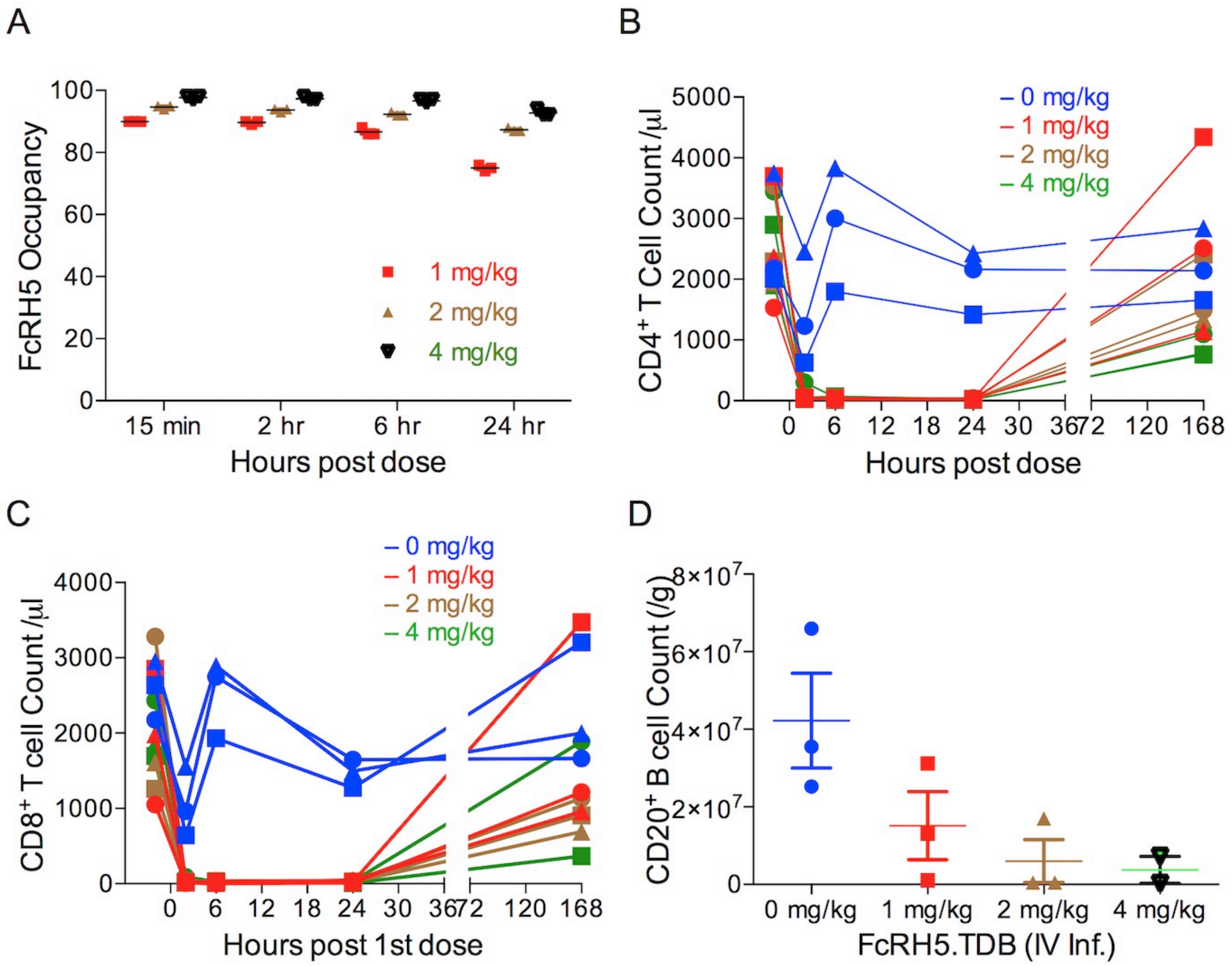

Figure S4. FcRH5 target occupancy, T cell dynamics ad depletion of B cells in mesenteric lymph nodes in cynomolgus monkey. Related to Figure 7. Four groups of animals after single dose intravenous administration of vehicle (blue), anti-FcRH5/CD3 at 1 mg/kg (red), 2 mg/kg (brown) or 4 mg/kg (green) were included in the graphs. **(A)** FcRH5 occupancy (RO) for anti-FcRH5/CD3 on circulating cyno B cells was calculated using a formula: % RO = C /(K_d + C) x 100. **(B)** The absolute count of $CD4^+$ T cells in peripheral blood. **(C)** The absolute count of $CD8^+$ T cells in peripheral blood. **(D)** Absolute counts of $CD20^+$ B cells in mesenteric lymph nodes after treatment. The plot is graphed as individual animals and the mean ± SEM.

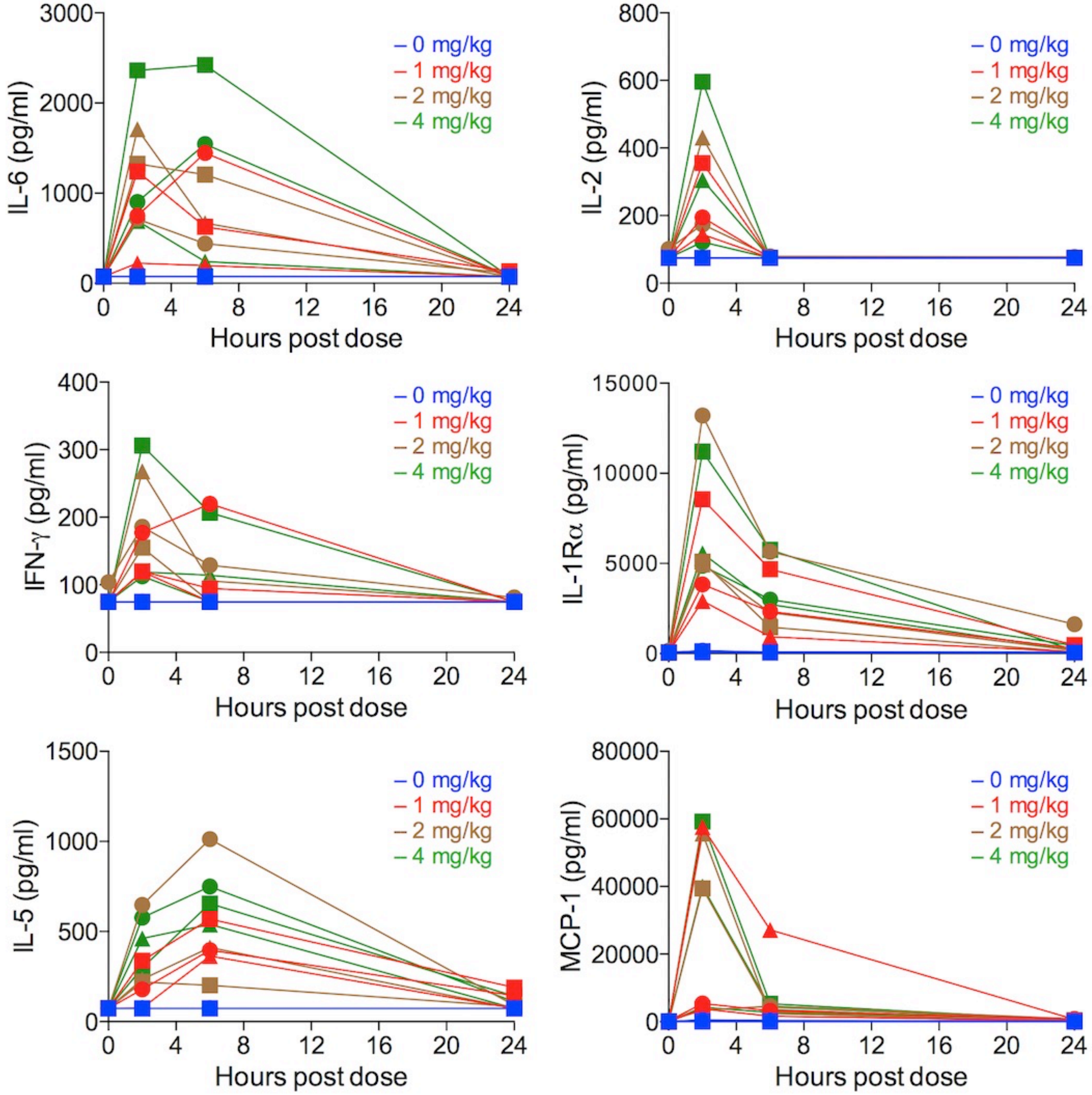

Figure S5. Anti-FcRH5/CD3 TDB induced transient cytokine release in cynomolgus monkey. **Related to Figure 7**. Four groups of animals after single dose intravenous administration of vehicle (blue), Anti-FcRH5/CD3 at 1 mg/kg (red), Anti-FcRH5/CD3 at 2 mg/kg (brown) and Anti-FcRH5/CD3 at 4 mg/kg (green) were included in the graphs.

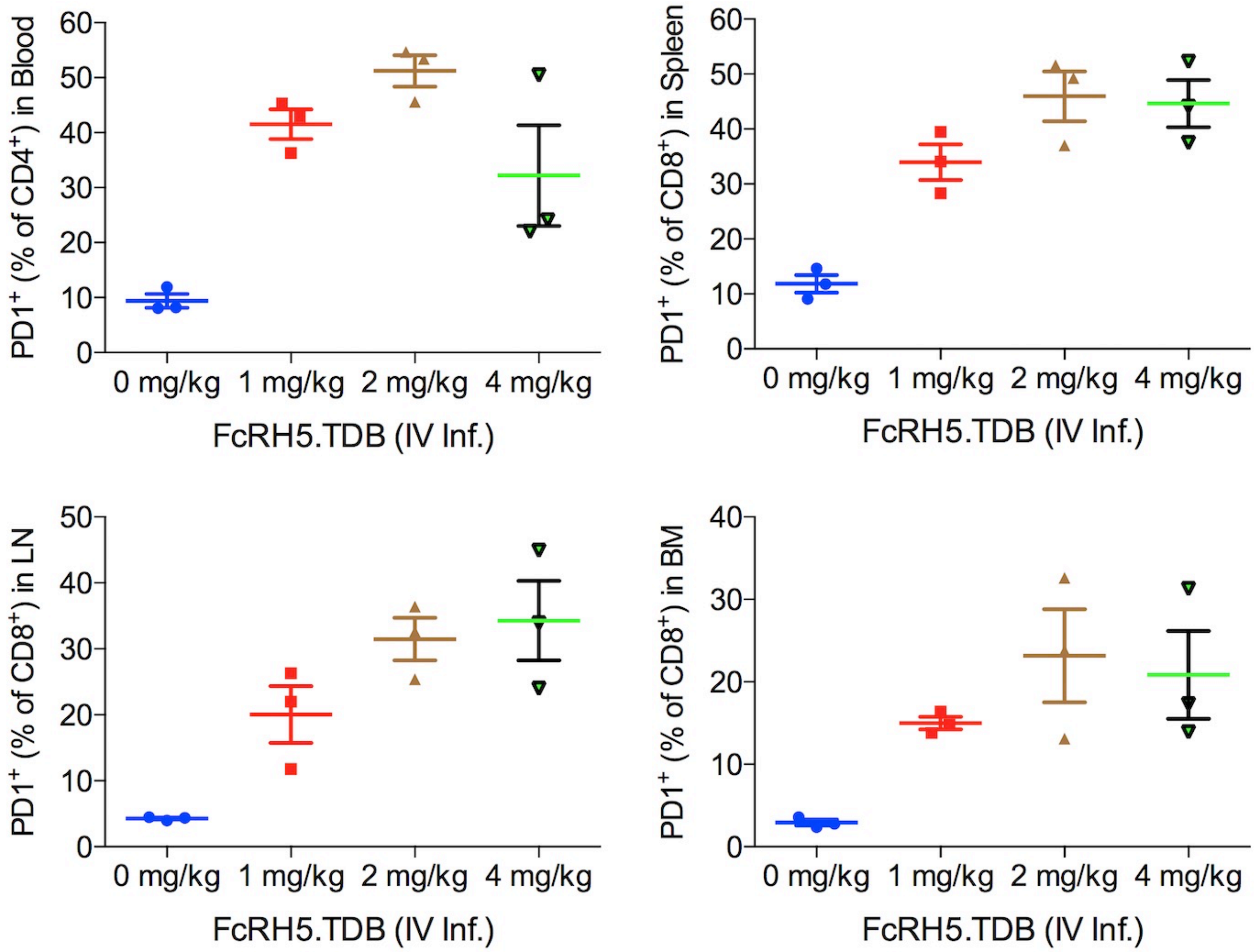

Figure S6. Anti-FcRH5/CD3 TDB induces PD-1 expression in cyno T cells in blood, spleen, lymph node and bone marrow. Related to Figure 8. PD-1 expression in $CD4^+$ and $CD8^+$ T cells was analyzed by flow cytometry 7 days after dosing anti-FcRH5/CD3 TDB or vehicle. Data are represented as the mean ± SEM.

Supplemental Experimental Procedures

RNA isolation, cDNA synthesis, and gene expression analysis

Total RNA was extracted from decalcified Formalin-fixed, paraffin-embedded (FFPE) bone marrow biopsy tissues collected from Multiple Myeloma patients. FFPE sections were deparaffinized, then lysate and RNA were prepared using the High Pure FFPET RNA Isolation Kit (Roche Applied Sciences, Indianapolis, IN) according to the manufacturer's protocol. Reverse transcription of complementary DNA (cDNA) and pre-amplification was generated in a single reaction using SuperScript® III Platinum® One-Step qRT-PCR Kit (ThermoFisher Scientific). Gene expression analysis was performed on the Fluidigm Biomark HD with custom Taqman assays (Applied Biosystems). The thermal cycling conditions are as follows: 1 cycle at 50˚C for 15 min, 1 cycle at 70˚C for 2 min, then 14 cycles at 95˚C for 15 sec and 60˚C for 4 min. Two reference genes, SDHA and VPS33B, were evaluated for each sample and used to calculate expression of FcRH5. Gene expression of FcRH5 was determined by using the delta Ct (dCt) method [$Ct_{\text{gene of interest}} – Ct_{\text{geometric mean of reference genes}}$].

Cytogenetic fluorescent in situ hybridization (FISH)

1q21 + copy control 1 FISH probe (BioCare Medical; previously CymogenDx, Concord, CA) was used to analyze 1q21 region. The 1q21 probe covers the chromosomal band 1q21.3 while the control probe is located in the peri-centromeric 1p12 region of chromosome 1. FISH analysis on formalin-fixed paraffin-embedded (FFPE) tissue was performed as described previously (Koeppen et al., 2014; O'Brien et al., 2008) with some modifications. After an overnight incubation at 56°C, the FFPE slides (5µ thick) were de-paraffinized in 3 washes of CitroSolv for 5 min each, dehydrated by two washes in alcohol and air-dried. Subsequently, the FFPE sections were incubated in a Pretreatment solution (Abbott Molecular) for 30 min at 80ºC and followed with a Protease II (Abbott Molecular) digestion prior to additional washes in water and a series of ethanol. Dried slides were then co-denatured (76°C for 6 min) with the probe set and were hybridized overnight at 37°C (ThermoBrite; Vysis, Downers Grove, IL). Post-hybridization washes and counter- staining were done in a manner similar to those previously described (Koeppen et al., 2014). The sections were viewed using fluorescence microscopes equipped with computer-driven imaging systems (BioView Duet, BioView Ltd., Israel). In the FFPE tissue samples, a minimum of 100 non-overlapping tumor cells from each sample was enumerated for the 1q21.3 (target) and 1p12 (control) locus. A tumor sample was identified as FISH positive when >20% of the tumor cells scored had 3 or more copies of the 1q21.3 locus.

huNSG/MOLP-2 mouse xenograft model

Since anti-CD3 antibodies do not cross-react with mouse CD3 we used a mouse model with reconstituted human immune systems ("humanized mice") to demonstrate in vivo anti myeloma activity of anti-FcRH5/CD3 TDB. Irradiated female NOD.Cg Prkdcscid Il2rgtm1Wjl/SzJ (common name NOD/scid gamma; NSG) mice were transplanted with CD34+ selected human hematopoietic stem cells to generate the humanized huNSG mice. huNSG mice were obtained from The Jackson Laboratory. 20 weeks post CD34+ cell transplantation huNSG mice were inoculated with 20 million MOLP-2 cells subcutaneously. MOLP-2 (American Type Culture Collection, Manassas, VA) is a human multiple myeloma cell line that express human FcRH5 endogenously and can be used as a xenograft tumor model. On the day of the cell inoculation, 5 animals were inoculated with 0.2 ml of MOLP2 tumor cells at a concentration of 100 million cells/ml, in HBSS/matrigel (1:1 ratio), subcutaneously in the right flank. As soon as the tumor volumes reached a volume range of 100-250 mm^3, animals were randomized into 2 groups and the first treatments were administered at that time (Day 0). All treatments were administered once a week for by intravenous (IV) tail vein injection. Tumors were measured 1-2 times per week with calipers for the duration of the study and animal body weights were recorded at least once a week. Clinical observations were performed twice per week to monitor the health of the animals. All in vivo experimental procedures conformed to the guiding principles of the American Physiology Society and were approved by Genentech's Institutional Animal Care and Use Committee. Only animals that appeared to be healthy and that were free of obvious abnormalities were used for the study.
